# Decoding mEos4b day‐long maturation and engineering fast‐maturing variants

**DOI:** 10.1002/pro.70234

**Published:** 2025-07-16

**Authors:** Arijit Maity, Oleksandr Glushonkov, Isabel Ayala, Pascale Tacnet, Jip Wulffelé, Philippe Frachet, Bernhard Brutscher, Dominique Bourgeois, Virgile Adam

**Affiliations:** ^1^ Univ. Grenoble Alpes, CEA, CNRS, Institut de Biologie Structurale Grenoble France; ^2^ Integrated Structural Biology Grenoble (ISBG), Université Grenoble Alpes, CNRS, CEA, EMBL Grenoble France

**Keywords:** chromophore, fluorescent proteins, maturation, NMR, protein engineering, spectroscopy, super resolution microscopy, X‐ray crystallography

## Abstract

The maturation speed of fluorescent proteins is a crucial parameter that influences cellular brightness, effective labeling efficiency, and temporal resolution in fluorescence microscopy. Green‐to‐red photoconvertible fluorescent proteins (PCFPs) used in pulse‐chase experiments and super‐resolution techniques such as Photoactivated Localization Microscopy (PALM), single‐particle‐tracking PALM (sptPALM), and Minimal Fluorescence Photon Fluxes Microscopy (MINFLUX) may be hampered by slow maturation. We systematically characterized the maturation speed of mEos‐derived PCFPs in *Escherichia coli* and found that, in contrast to pcStar and mEosEM, several variants such as mEos2, mEos3.1, mEos3.2, and mEos4b mature extremely slowly. Strikingly, the oxidation step in these PCFPs is fast and not rate‐limiting. Through a structure‐guided mutagenesis strategy, we developed a strategy to reduce the day‐long maturation time of mEos4b by nearly two orders of magnitude without significantly impacting its molecular brightness and photophysical performance under single‐molecule imaging conditions.

## 1 INTRODUCTION

Fluorescent proteins have revolutionized the field of cell biology, serving as indispensable tools for visualizing and tracking cellular processes. Their ability to act as non‐invasive markers has significantly contributed to our understanding of complex biological phenomena. However, a persistent challenge for the utility of fluorescent proteins is the potentially slow maturation of their chromophore. The rate of chromophore maturation is a critical factor that affects various aspects of cellular imaging, including apparent brightness, effective labeling efficiency, and temporal resolution. Slow maturation may also result in artifacts in FRET measurements (Liu et al., 2018; Sreenivasan et al., 2020) and the toxic accumulation of hydrogen peroxide in cells (Ganini et al., 2017). Photoconvertible fluorescent proteins (PCFPs) are commonly employed in super‐resolution microscopy techniques such as Photo‐Activated Localization Microscopy (PALM), single‐particle‐tracking PALM (sptPALM) and more recently MINFLUX (Balzarotti et al., 2017). Slow maturation can be a notable drawback since it may hamper the successful visualization and analysis of cellular structures at enhanced resolutions, notably in live cells. In this work, we present a comprehensive investigation of the maturation speed of PCFPs from the mEos family and propose strategies to considerably reduce the maturation time of the monomeric, bright, and fixation‐resistant mEos4b variant (Paez‐Segala et al., 2015).

**FIGURE 1:**
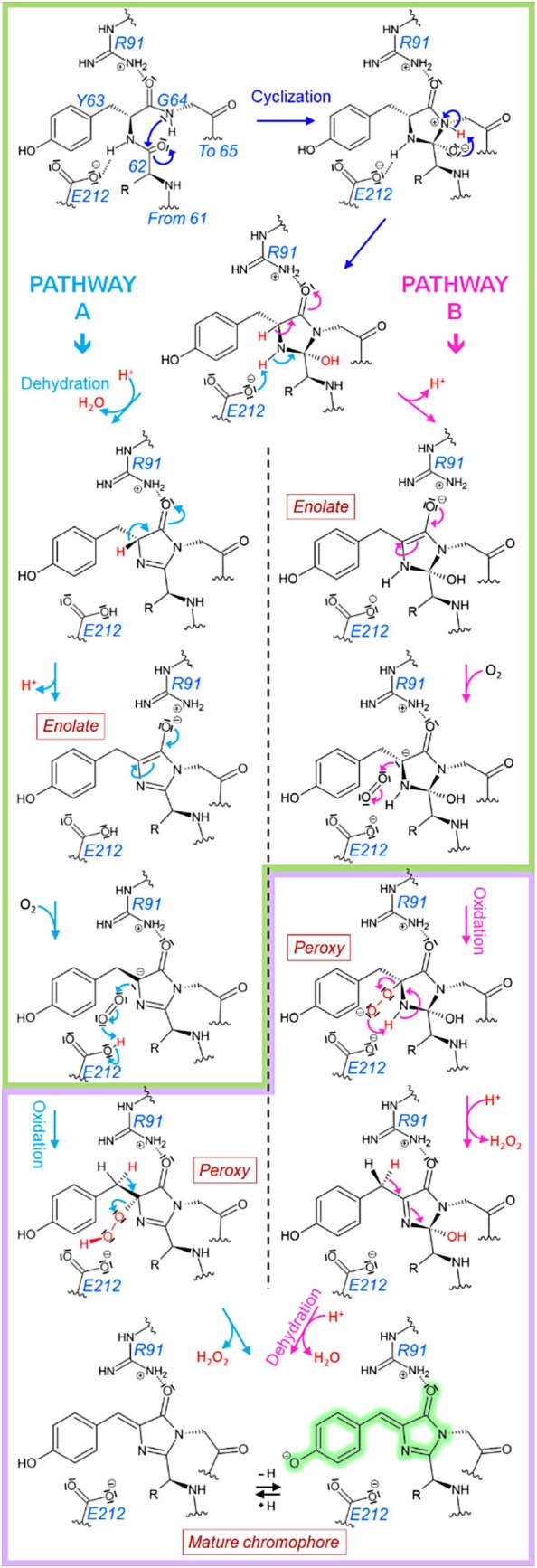
Chemical reaction steps of maturation from the pre‐cyclized to the mature chromophore in green FPs. The first residue of the chromophore is variable (His 62 in Eos) and represented by “R”. The numbering is that of the Eos family. Following the cyclization (blue), two possible pathways have been proposed for which reactions are putative. In the first pathway (cyan), a dehydration step followed by an oxidation step involving molecular oxygen, results in the elimination of hydrogen peroxide. In the alternative pathway (pink), these steps are reversed. Both pathways result in the formation of double bonds, allowing the establishment of a conjugated system in the mature chromophore. Leaving groups are shown in red. The oxygen‐independent steps are framed in green while the oxygen‐dependent steps are framed in purple.

The mechanism by which the chromophore of fluorescent proteins matures has been widely studied, but remains not fully understood. After protein folding, the carbonyl group of the non‐conserved first amino acid in the chromophoric triad (histidine in green‐to‐red PCFPs, H62 in Eos numbering) and the amide function of the third amino acid (conserved glycine, G64 in Eos numbering) come into proximity, as a result of the prototypical kinked central α‐helix of the FP scaffold (Barondeau et al., 2003). In this orientation, a nucleophilic attack of the nitrogen from G64 (Wachter, 2007; Wood et al., 2005) on the carbonyl of residue 62 leads to the formation of a covalent bond in a process called cyclization. This cyclization step forms an initial intermediate with an imidazolidine ring. The subsequent reactions involve the formation of double bonds through an oxidation step and a dehydration step. Depending on the order of these two steps, two competing models have been proposed (Figure 1).

According to the first model (Figure 1, cyan arrows) the dehydration step precedes the oxidation step (Auhim et al., 2021; Barondeau et al., 2006; Cubitt et al., 1995; Grigorenko et al., 2017; Heim et al., 1994; Pletneva et al., 2010, 2021; Reid & Flynn, 1997; Tsien, 1998). Elimination of a hydroxyl group and one proton results in the formation of a first double bond in an imidazolinone ring. Molecular oxygen attack on the chromophore then results in a peroxy intermediate, leading to the elimination of hydrogen peroxide and the formation of a second double bond establishing conjugation between the hydroxybenzylidene and imidazolinone groups.

According to the second model (Figure 1, pink arrows), the oxidation step precedes the dehydration step (Ma et al., 2017; Pouwels et al., 2008; Rosenow et al., 2004; Zhang et al., 2006). Molecular oxygen attack results in a peroxy intermediate, and following the elimination of hydrogen peroxide, a new hydroxyl imidazolinone intermediate is formed. Dehydration then leads to the mature chromophore. The possibility of both pathways acting in parallel has also been discussed (Craggs, 2009).

Regardless of the pathway, these maturation steps are facilitated by the chromophore's environment, notably the strictly conserved amino acids glutamate 212 (222 in EGFP) and arginine 91 (96 in EGFP). E212 functions as a general base, facilitating deprotonation of the chromophore G64 or abstracting a proton from the α‐carbon of Y66, possibly mediated via a water molecule (Lemay et al., 2008; Sniegowski, Lappe, et al., 2005; Wachter, 2007).

Similarly, R91 promotes electrophilic catalysis through its interaction with the carbonyl group of G64, notably by stabilizing the enolate intermediate, key to chromophore cyclization (Barondeau et al., 2006; Ma et al., 2010; Ma et al., 2015; Sniegowski, Phail, & Wachter, 2005; Tsien, 1998; Wood et al., 2005). Mutation of these conserved residues is not well tolerated and, for example, the mutation of glutamate to glutamine slows down the maturation process at physiological pH and accelerates it at very basic pH (Sniegowski, Lappe, et al., 2005). The exchange of the arginine with a different amino acid (except lysine) was reported to drastically slow down maturation kinetics (Sniegowski, Phail, & Wachter, 2005; Wood et al., 2005) up to several months (Barondeau et al., 2003; Sniegowski, Lappe, et al., 2005).

Furthermore, the precise positioning and interaction patterns of E212 and R91 within the FP scaffold may affect the maturation rate. To date, mechanistic studies on FP maturation have only been conducted on hydrozoan GFP variants. The emerging picture is that the rate‐limiting step in maturation is the oxidation step (Cubitt et al., 1995; Heim et al., 1994; Nagai et al., 2002; Reid & Flynn, 1997; Zhang et al., 2006), whereas cyclization is a fast process (Reid & Flynn, 1997). However, whether this scenario also applies to FPs of anthozoan origin remains unknown.

Recent advancements have seen the emergence of FP variants with faster maturing chromophores compared to their predecessors, such as Venus (Nagai et al., 2002), DsRed‐express (Bevis & Glick, 2002), and DsRed‐express2 (Strack et al., 2008), mScarlet3 (Gadella et al., 2023), Kohinoor2.0 (Wazawa et al., 2021), and mStayGold (Ando et al., 2024). Since a general rationale for the improved maturation kinetics is missing, the mutagenesis strategy employed for these FPs cannot be translated directly to improve the maturation speed of other FPs. It is also important to note that maturation rates reported in the literature for single FPs (e.g., mNeonGreen, Clover, mStayGold, mBaojin, or Ds‐Red express variants) (Ando et al., 2024; Balleza et al., 2018; Bevis & Glick, 2002; Shaner et al., 2013; Zhang et al., 2024) vary significantly. The exact definition of maturation kinetics also differs between studies, and the techniques employed to measure maturation rates are diverse (as it is the case for other parameters such as quantum yield or extinction coefficient (Nienhaus & Nienhaus, 2021)), leading to discrepancies in the reported values. For example, refolding time after chemical denaturation is sometimes misreported as the maturation time, although these represent distinct processes (Zhang et al., 2012). Consequently, data available in repositories such as FPbase (Lambert, 2023) may not always be reliable. Balleza et al. have highlighted the need for a rigorous study of maturation rates in FPs (Balleza et al., 2018), but a systematic investigation of PCFP maturation rates has not been carried out until now.

When studying mEos4b by Nuclear Magnetic Resonance (NMR) spectroscopy in the frame of our recent work (Maity et al., 2024), we discovered that this PCFP exhibits exceptionally slow maturation. As this unfavorable property has not been reported previously in the literature, we undertook a detailed investigation of maturation kinetics in proteins of the mEos family. To this aim, we devised a well‐defined methodology to measure the apparent maturation rate (from folding to appearance of fluorescence signal, i.e., the parameter of practical importance for imaging applications) of PCFPs. Moreover, we separately measured the rate of the oxidation (and possibly post‐oxidation) step.

Our measurements revealed that the most likely rate‐limiting step for mEos4b maturation is the cyclization, which is contrary to previous reports on hydrozoan FPs, where oxidation was shown to be the rate‐limiting step (Guerra et al., 2022; Nagai et al., 2002; Pouwels et al., 2008). We also found that the two most recent members of the mEos family: pcStar (Zhang et al., 2020) and mEosEM (Fu et al., 2020), exhibit significantly faster maturation than older variants. To better understand the rationale behind these differences, we identified the key mutations involved. With the help of crystallographic structures, AlphaFold calculations (Abramson et al., 2024) and molecular dynamics (MD) simulations (Liebschner et al., 2019), we performed site‐directed mutagenesis studies on mEos4b. Among the variants generated were two double mutants named mEos4Fast1 and mEos4Fast2, which exhibited markedly accelerated maturation kinetics compared to their parent while retaining other biochemical and photophysical properties. Finally, to validate the practical utility of faster mutants, we applied the engineered variants for cellular imaging in both prokaryotic and eukaryotic cells.

## 2 RESULTS

### 2.1 NMR insights and comparative maturation kinetics

**FIGURE 2:**
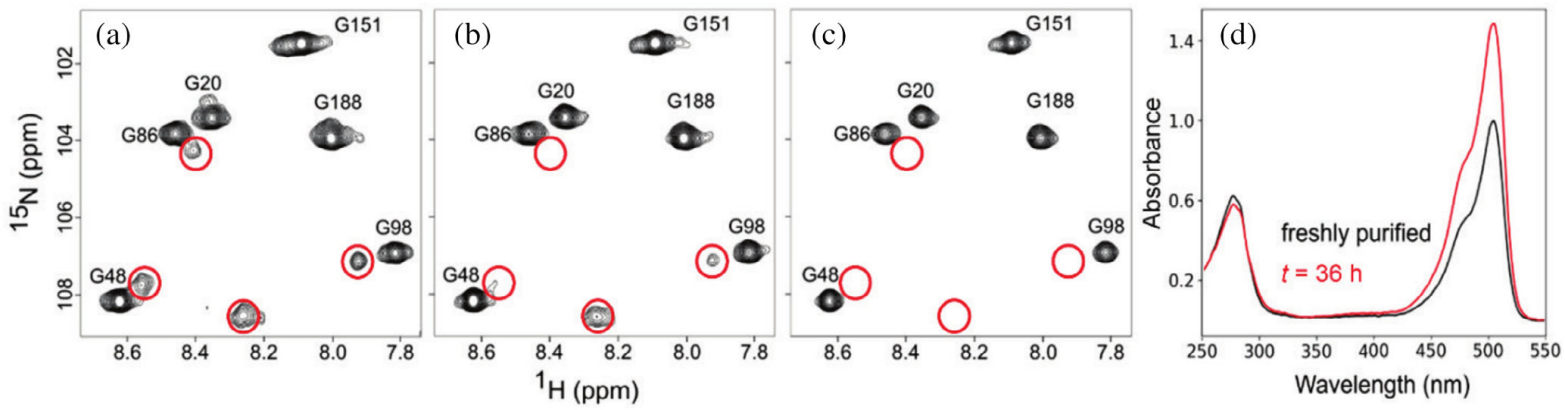
Slow chromophore maturation kinetics observed in NMR and UV–Vis spectroscopy at 35°C. (a–c) Small region of NMR ^1^H‐^15^N spectra of mEos4b acquired (a) just after protein production and purification, and after (b) 8 h and (c) 36 h in the NMR magnet. NMR peaks disappearing during maturation of the chromophore are highlighted by red circles, while visible green state peaks are annotated by their residue number and type. Full NMR spectra are provided in Figure S1a. (d) Comparison of UV–Vis spectra of freshly purified (black) sample and after 36 h (red) in the NMR magnet. Experiments were performed under ambient atmospheric conditions (~20% O_2_).

During our recent study of mEos4b photophysics by solution NMR spectroscopy (Maity et al., 2024), we observed an exceptionally long maturation process for this PCFP. NMR ^1^H‐^15^N spectra of mEos4b, acquired immediately after protein production and purification, and up to 36 h later while kept in the NMR magnet at 35°C, revealed the gradual disappearance of a set of NMR peaks corresponding to a minor fraction (~25%) of a well‐folded species. This species was tentatively assigned to proteins containing an incompletely matured chromophore (Figures 2a–c and S1a). Concurrently, the intensity of NMR peaks corresponding to residues in the green fluorescent state, which do not overlap with the minor peak species (e.g., G48, G86, and G98) increased over the course of the kinetic experiment.

A comparison of the UV–Vis absorption spectra of a freshly purified sample recorded before and immediately after the NMR experiment (after 36 h) revealed a significantly increased absorption band at 504 nm, providing evidence for incomplete chromophore maturation at the start of NMR data collection (Figure 2d).

The evolution of the NMR signal intensity thus provided a measure of apparent maturation kinetics. Fitting these NMR data to a monoexponential kinetic model yielded an apparent maturation (i.e., observable fluorescence build‐up from folding to fully mature chromophore) time constant of about 1200 min (Figure S1b) at 35°C, that is more than an order of magnitude slower than previously reported values for members of the mEos family, namely mEosFP, mEos2, mEos3.1, and mEos3.2 (Marathe et al., 2020; Zhang et al., 2012). However, these studies essentially reported different parameters as the maturation time.

While Zhang et al. reported the refolding time following a chemical denaturation, Marathe et al. reported the time needed for the oxidation (or post oxidation events, depending on the maturation pathway, see Section 3).

**FIGURE 3:**
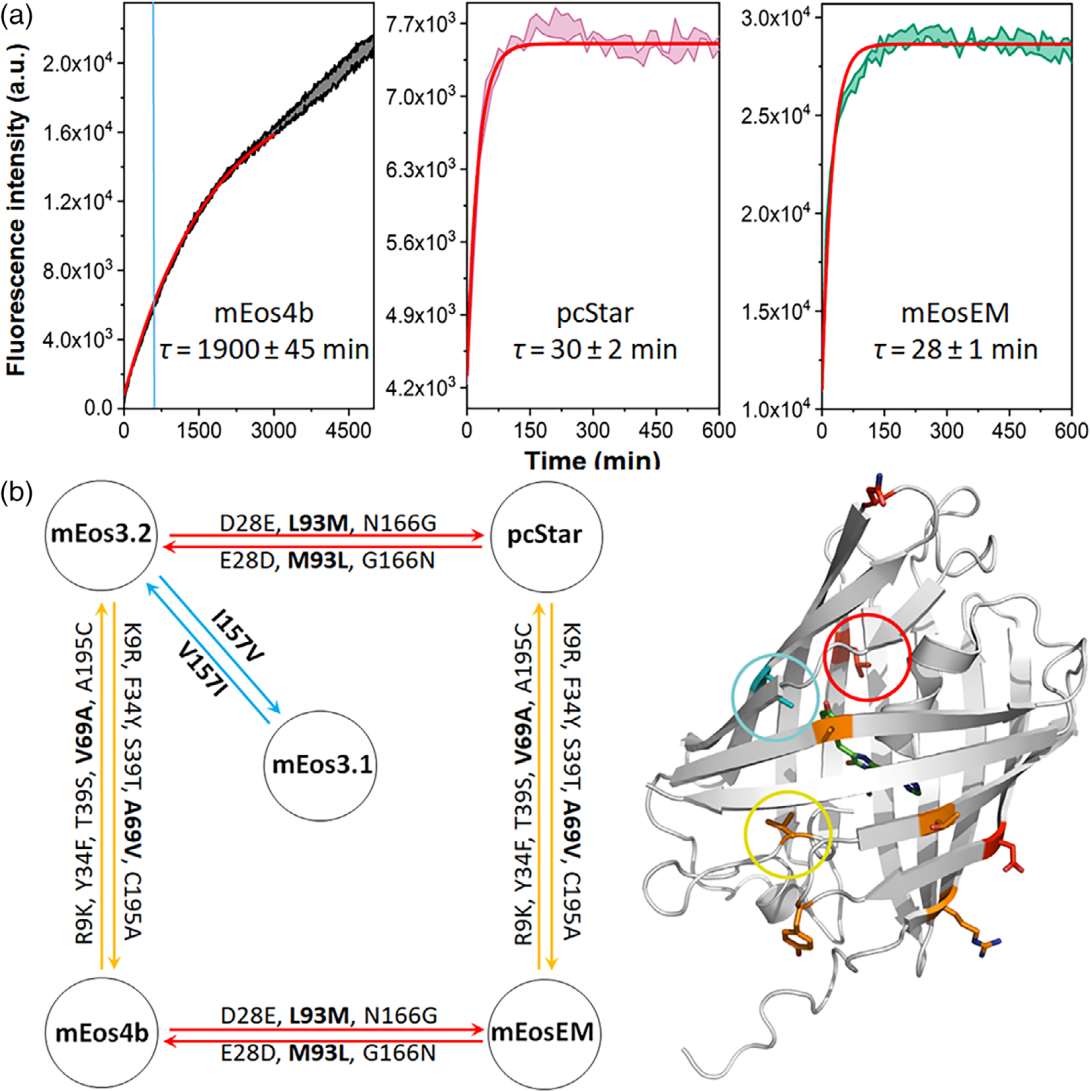
(a) Apparent maturation rates measured by fluorescence increase in bacteria of mEos4b and the two latest mEos derivatives pcStar and mEosEM. All fluorescence measurements were performed at 25°C. For mEos4b the total acquisition time was longer than represented and the vertical blue line represents the time window that was used for pcStar and mEosEM. (b) Mutation map of mEos4b with its closest relatives, namely mEos3.1, mEos3.2, pcStar, and mEosEM. All mutations are mapped on the crystal structure of mEos4b (PDB: 6GOY), with the mutation sites inside the barrel circled and shown in bold. Experiments were performed under ambient atmospheric conditions (~20% O_2_).

We therefore measured the maturation kinetics of mEos4b and other members of the mEos family using a fluorescence‐based assay in vitro. At 25°C, the fluorescence build‐up of mEos4b pointed to an extremely slow maturation compared to the more recent pcStar and mEosEM (Figure 3a), in line with our NMR data. The fluorescence did not reach a plateau even after 5000 min, suggesting that maturation was still incomplete. Furthermore, the fluorescence curve clearly indicated the presence of at least two populations that matured at different rates. A monoexponential fit to the early fluorescence increase (*τ* = 1900 ± 45 min) captured the main maturation phase. Later deviations, likely due to drift or slow processes, impaired full‐curve fitting without altering the overall trend (see Appendix S1).

**FIGURE 4:**
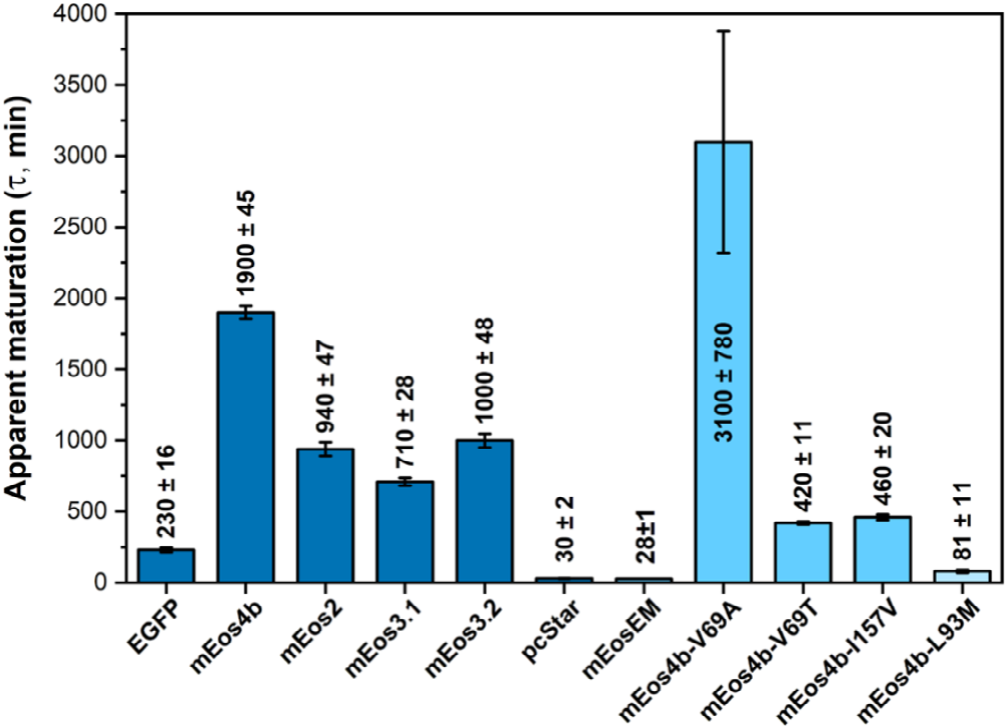
Time constants for the fast phase of apparent maturation kinetics (folding + cyclization + dehydration + oxidation) for EGFP (control) and mEos4b and its ancestors (dark blue) as compared to single mEos4b variants (light blue). Error bars are standard deviations of time constants extracted from three separate measurements. Experiments were performed under ambient atmospheric conditions (~20% O_2_).

The data also revealed that despite very similar sequences (Figure 3b), a significantly slower maturation rate for mEos4b was observed as compared to its ancestors mEos2, mEos3.1, and mEos3.2 (Figures 4 and S2). However, all these variants showed maturation times exceeding 11 h, and similar to mEos4b, some of the variants, such as mEos2 and mEos3.2, clearly showed multiple populations maturing at different rates (Figure S2). Only the recent mEos variants, pcStar, and mEosEM, exhibited markedly accelerated maturation with time constants of the order of 30 min. For pcStar and mEosEM, the entire datasets could be well fitted with a monoexponential function.

### 2.2 Residue at position 93 is key for maturation

In light of the several orders of magnitude differences in maturation rates observed among various mEos relatives, we conducted a detailed comparison of their primary sequences. Figure 3b shows the sequence relationship between mEos4b, mEos3.1, mEos3.2, pcStar, and mEosEM.

When compared to mEos4b and mEosEM, most of the differences in the other three proteins are located outside the β‐barrel. While long‐range effects have been previously shown to affect properties of fluorescent proteins (Gadella et al., 2023; Kim et al., 2013), they are difficult to predict.

Therefore, we primarily focused on residues with side chains pointing towards the interior of the β‐barrel, as depicted in Figure 3b, specifically positions 69, 93, and 157. As illustrated in the same figure, the only difference between mEos3.1 and mEos3.2 occurs at position 157, which is a valine in mEos3.1 and an isoleucine in mEos3.2. Yet, this single substitution from valine to isoleucine increases the maturation time of mEos3.2 by ~40% (Figure 4). Similarly, we hypothesized that the L93M mutation, which distinguishes mEos3.2 from pcStar and mEos4b from mEosEM, could be pivotal in the significant reduction in maturation time observed for pcStar and mEosEM. Additionally, we speculated that the A69V mutation could also cause a near doubling of maturation time in mEos4b as compared to mEos3.2.

To test these hypotheses, we generated the I157V, L93M, and V69A substitutions in mEos4b and measured their maturation kinetics. Additionally, we generated the V69T variant to test a charged residue that we previously identified as important to modify the microenvironment of the chromophore (Berardozzi et al., 2016). Our measurements showed that, while the mutation V69A further slowed down the maturation kinetics of mEos4b, the three other single‐point mutants improved it. Despite this, the V69T and I157V mutations were still unsatisfactory, with maturation time constants exceeding 400 min. In contrast, the L93M mutant showed a drastically decreased apparent maturation time of approximately 80 min, a more than 20‐fold improvement relative to the mEos4b parent (Figures 4 and S3a) and better than any other L93 substitution we have tested (Table S1).

Despite this improvement, the maturation rate of mEos4b‐L93M remained approximately three‐fold slower compared to that of pcStar or mEosEM (Figure 4), both of which also have a methionine at position 93 (Figure 3b). Hence, we aimed to identify additional mutations that would further enhance maturation rates without compromising other photophysical properties, particularly in the photoconverted red‐state (the state of interest in most experiments employing PCFPs).

### 2.3 The crystal structure of mEos4b‐L93M reveals a water cavity near the chromophore

As a first step towards further improvements, we sought to understand the rationale behind the observed accelerated maturation for mEos4b‐L93M. To this end, we solved the structure of mEos4b‐L93M (PDB: 9GVR) by X‐ray crystallography and compared it with the previously published structure of mEos4b (PDB ID: 6GOY) (De Zitter et al., 2019).

**FIGURE 5:**
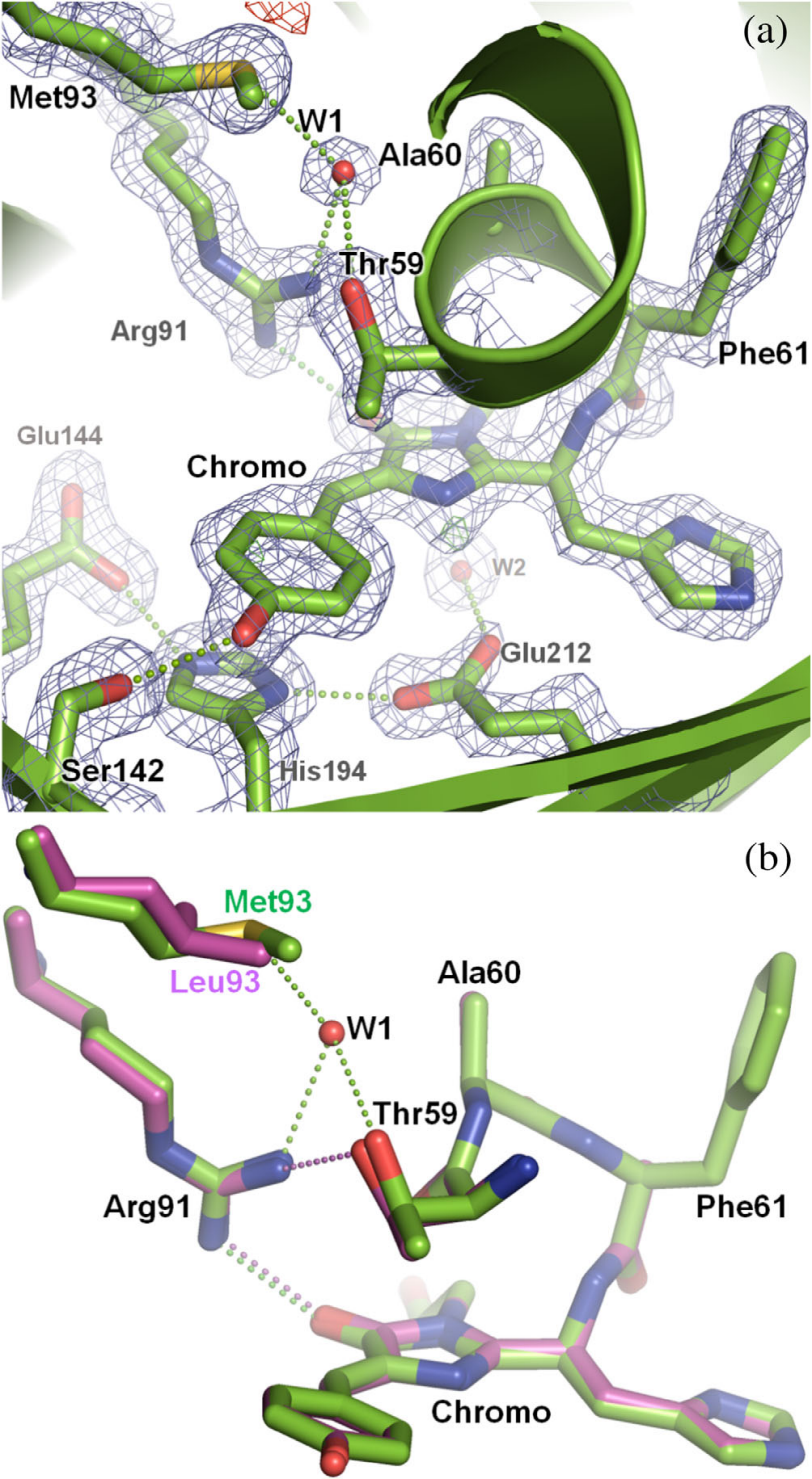
Crystallographic structure of mEos4b‐L93M. (a) The chromophore and its environment are shown with green carbons, contoured by 2*F*_obs_ − *F*_calc_ electron density map (1.5 σ) and *F*_obs_ − *F*_calc_ difference electron density map (±3.0*σ*). The mutation L93M allows the stabilization of a new water molecule, labeled W1. Hydrogen bonds (≤3.3 Å) are depicted as green and purple dashed lines for mEos4b‐L93M and mEos4b, respectively. (b) Compared to its parent mEos4b, shown with purple carbons, the interaction between W1 and R91 relocalizes this arginine relative to the chromophore and breaks its hydrogen bond with the side chain of T59 (from 3 to 3.6 Å).

The only significant difference (beside the L93M mutation) was the presence of an extra water molecule in the vicinity of the chromophore, which is well ordered as evidenced by the clear electron density map at this position (Figure 5a). This water molecule (W1 hereafter) is within hydrogen‐bonding distance of the sulfur atom of M93 and the hydroxyl group of T59. It also interacts with the side chain guanidinium group of R91, thereby providing a partner that modulates the interaction of R91 with the chromophore and the hydrogen bond with the side chain of T59 observed in mEos4b (Figure 5b).

### 2.4 Fast‐maturing variants mEos4Fast1 and mEos4Fast2

Aligning the 3D structures of the fast‐maturing and bright fluorescent proteins mNeonGreen (PDB: 5LTR) and mScarlet3 (PDB: 7ZCT) with that of mEos4b (PDB: 6GOY) (Figure S4), we observed a hydrogen‐bonding (H‐bond) interaction between the side chains at position 60 and R91 in the two fast‐maturing proteins. This H‐bond cannot be formed in mEos4b, which harbors an alanine at position 60.

We hypothesized that this interaction could play a role in positioning the key residue R91 to facilitate rapid maturation. In addition, an exploration of all fluorescent proteins with reported maturation times in the fluorescent proteins database (Lambert, 2023) (FPbase, https://www.fpbase.org/) revealed that the position equivalent to A60 is commonly occupied by glutamine, aspartate, serine, or threonine (Figure S5).

**FIGURE 6:**
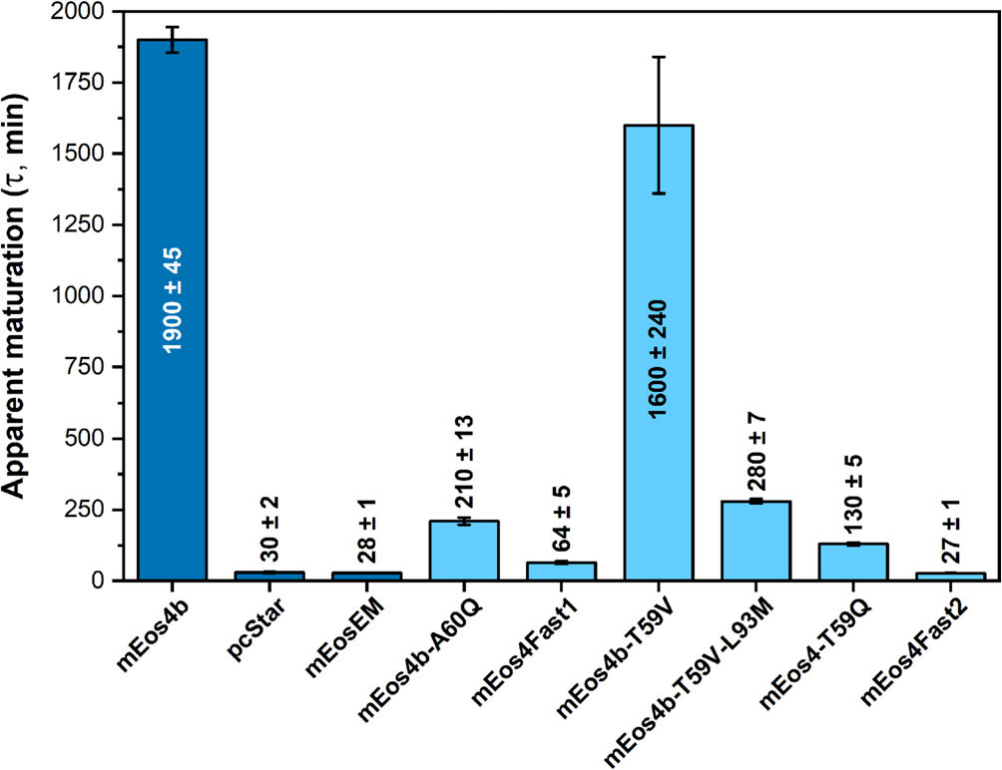
Comparison of time constants of apparent maturation (folding + cyclization + dehydration + oxidation) for mEos4b and selected derivatives (dark blue) as compared to variants that led to the creation of mEos4Fast1 and mEos4Fast2 (light blue). Error bars are standard deviations of time constants extracted from three separate measurements. Experiments were performed under ambient atmospheric conditions (~20% O_2_).

Based on our structural data, we speculated that this side chain may occupy the space of the water molecule observed for mEos4b‐L93M and provide a hydrogen bonding partner to R91. Thus, we generated and tested mEos4b mutations of A60 to either aspartate, glutamine, serine, or threonine. In addition, we also mutated A60 to histidine and proline, as in the fast‐maturing FPs mNeonGreen (Shaner et al., 2013) and Gamillus (Shinoda et al., 2018), respectively. Finally, we tested the A60K mutation to explore if a long, basic but flexible side chain could still adapt. Among these mutants, only the A60Q variant yielded a bright fluorescent protein with a significantly (~9‐fold) improved maturation speed compared to the mEos4b parent (Figure 6).

Introducing the single A60Q mutation resulted in maturation times of 209 ± 13 min for mEos4b‐A60Q and 64 ± 5 min for mEos4b‐A60Q‐L93M. We named this fast‐maturing double mutant mEos4Fast1. Despite our efforts, we were unable to crystallize mEos4Fast1, so we used AlphaFold 3 (Abramson et al., 2024) and MD simulations in the Phenix package (Liebschner et al., 2019) to generate structural models of this variant (Figure S6). In both computed structural models, the amide group of Q60 replaces the water molecule W1 observed in the mEos4b‐L93M structure, leading to a similar repositioning of the R91 side chain relative to T59. These observations rely mostly on computational modeling and should be considered speculative, pending further experimental confirmation. We also explored a glutamine mutation at position 59, where T59 forms a hydrogen bond with either the water molecule or the guanidinium group of R91 in the mEos4b‐L93M and mEos4Fast1 mutants.

We prepared the two mutants, mEos4b‐T59Q and mEos4b‐L93M‐T59Q. In addition, we generated mEos4b‐T59V and mEos4b‐L93M‐T59V as negative controls since valine is isosteric with threonine but lacks a polar side chain. As expected, mEos4b‐T59V and mEos4b‐T59V‐L93M exhibited slow maturation (Figure 6).

In contrast, the T59Q mutation significantly improved the apparent maturation time, particularly in the variant mEos4b‐T59Q‐L93M, which we renamed mEos4Fast2. This protein outperforms any other tested mEos variants and challenges the very fast‐maturing mEosEM and pcStar (Figure 6). We subsequently proceeded with the biochemical and photophysical characterization of mEos4Fast1 and mEos4Fast2.

### 2.5 Photophysical and biochemical properties of the new mutants

To investigate whether our fast‐maturing mEos4Fast1 and mEos4Fast2 variants retained the photophysical and biochemical properties of their mEos4b parent, we compared the in vitro fluorescence properties of mEos4b‐L93M, mEos4Fast1, and mEos4Fast2 with those of the latest mEos variants, namely mEos4b, pcStar, and mEosEM (Table 1).

**TABLE 1:** Photophysical parameters of the members of the mEos family in their green (G) and red (R) species at pH 7.5 measured at the ensemble level.

Protein	*λ*_ex,max_ (nm)	*λ*_em,max_ (nm)	*ε* (M^−1^ cm^−1^)	QY	Bright. (×10^3^)	pKa
mEos4b (G)	504	518	97,970	0.67	66	5.5^a^
mEos4b (R)	569	580	56,130	0.76	43	5.8^a^
pcStar (G)	504	516	80,910	0.61	50	5.5^a^
pcStar (R)	569	580	47,940	0.42	20	6.1^a^
mEosEM (G)	504	515	88,280	0.69	61	5.6^a^
mEosEM (R)	570	581	51,090	0.48	24	6.5^a^
mEos4b‐L93M (G)	504	515	77,120	0.68	52	5.6
mEos4b‐L93M (R)	569	580	57,200	0.63	36	6.3
mEos4Fast1 (G)	505	517	93,170	0.78	73	5.6
mEos4Fast1 (R)	570	580	58,450	0.73	43	6.7
mEos4Fast2 (G)	499	513	78,020	0.82	64	4.9
mEos4Fast2 (R)	565	580	43,290	0.54	23	5.4

*Note*: Brightness (bright.) is the product of the extinction coefficient (*ε*) and fluorescence quantum yield (QY).

^a^ All parameters were measured by us except for the six pKa values that were extracted from FPBase.

At the ensemble level, both mEos4Fast1 and mEos4Fast2 exhibit brightness in their green fluorescent form comparable to mEos4b and mEosEM, and significantly higher than pcStar. In its red form, mEos4Fast1 is as bright as mEos4b and significantly brighter than pcStar and mEosEM, while for red mEos4Fast2 similar values are found. Of note, mEos4Fast2 features a surprisingly low pKa both in its green and red forms. A low pKa is advantageous for ensemble brightness in a cellular context, notably in acidic compartments, but can also be problematic for green‐to‐red photoconversion as it requires more phototoxic 405‐nm light to excite the neutral chromophore. While green chromophores of all studied PCFPs are nearly entirely in their anionic (fluorescent) form at physiological pH (7.2), the red chromophores of pcStar and mEosEM are 87.0% and 76.0% in the anionic form, respectively, compared to 97.5% for mEos4Fast2.

At the single‐molecule level, mEos4b, mEos4Fast1, and mEos4Fast2 were compared for their blinking propensities, on‐times, off‐times, photon budget, and photostability in vitro by embedding in polyacrylamide gel (Figure S7). The proteins were essentially indistinguishable in terms of these properties, although we noticed that red mEos4Fast2 was slightly less bright at the single molecule level and exhibited slightly shorter on‐times and longer off‐times than the other variants. This possibly suggests facilitated cis–trans isomerization of the mEos4Fast2 chromophore as a source of blinking. Overall, we concluded that the faster maturation of mEos4Fast1 and mEos4Fast2 did not degrade the excellent single‐molecule photophysical properties of mEos4b.

To confirm that the introduced mutations did not alter the oligomeric state of the protein, we analyzed mEos4b, mEos4Fast1, and mEos4Fast2 using size exclusion chromatography coupled with multi angle light scattering (SEC‐MALS). All three proteins eluted as a single predominant peak corresponding to the monomeric molecular weight, with monomeric fractions of 95.0%, 99.5%, and 99.7%, respectively. These results indicate that engineering mEos4b to enhance maturation speed does not compromise its monomeric behavior.

Finally, we ensured that our fast‐maturing mEos4b variants retained their properties in common fixation reagents used in cell imaging, such as paraformaldehyde (PFA) and glutaraldehyde (GA) (Osuga et al., 2021) as well as in contrast reagents in electron microscopy such as osmium tetroxide (OsO_4_). While mEos4Fast1 showed moderate stability in the presence of PFA and GA, mEos4Fast2 emerged as the best fluorescent protein we tested, retaining 70%–80% of its fluorescence after fixation (Figure S8a). In the presence of OsO_4_, mEos4Fast1 retained a significant fraction of its fluorescence, performing slightly below mEos4b and mEosEM (the most resistant) and substantially better than EGFP. In contrast, mEos4Fast2, despite its excellent performance in PFA fixation, lost most of its fluorescence upon OsO_4_ exposure (Figure S8b).

### 2.6 Effect of faster maturation on apparent cellular brightness

The apparent cellular brightness and maturation speed of mEos4Fast1 and mEos4Fast2 were compared to those of their parent mEos4b in prokaryotic and eukaryotic cells. For prokaryote expression, cells were grown, plated and imaged regularly in fluorescence mode. After IPTG induction, a striking difference in green fluorescence signal was observed between the two fast‐maturing variants and mEos4b, with mEos4Fast2 becoming quickly brighter than mEos4Fast1, which in turn was significantly brighter than mEos4b (Figure S9). For eukaryote expression, U2OS cells were transfected with the bicistronic constructs mEos4b‐2a‐mCherry, mEos4Fast1‐2a‐mCherry, or mEos4Fast2‐2a‐mCherry. mCherry, connected to the target protein via the small viral T2A self‐cleavable linker, served to standardize the different transfection/expression levels in different cells.

**FIGURE 7:**
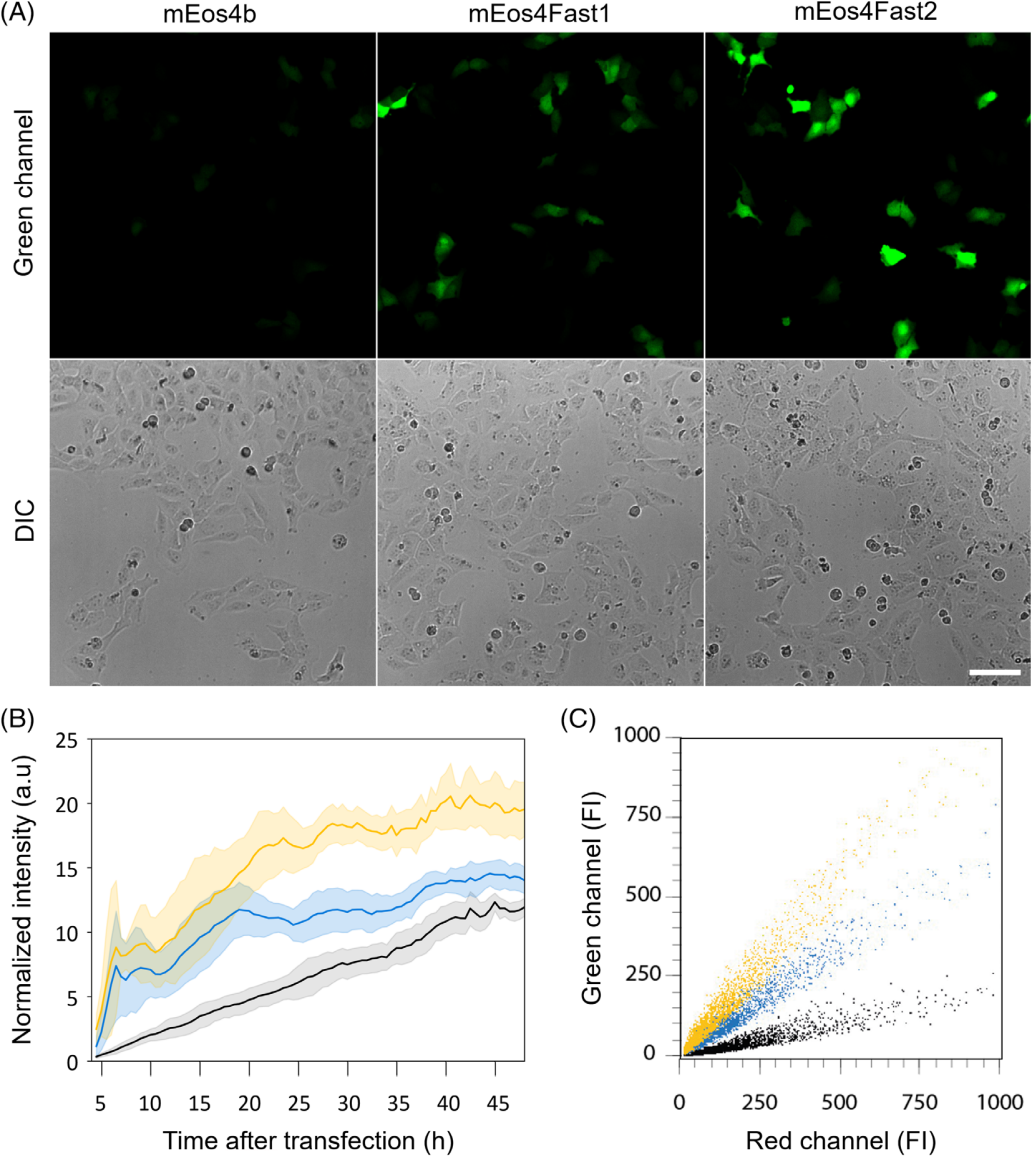
Maturation of mEos4b, mEos4Fast1, and mEos4Fast2 in bicistronic constructs with mCherry in U2OS cells. (a) Representative microscopic images of live U2OS cells transfected with each construct and observed 16 h after transfection. The green channel is normalized by the red channel of mCherry and each field of view contains a comparable number of cells as testified by images recorded in the DIC channel. (b) Mean fluorescence signal evolution for mEos4b (black), mEos4Fast1 (blue), and mEos4Fast2 (yellow) from live U2OS cells in five different field of views (*n* = 5). The green channel is normalized by the signal of mCherry at the end of the kinetics. (c) Flow cytometry analysis of cells 16 h after transfection (10,000 cells per construct). Dot plot of mEos4 (green) versus mCherry (red) fluorescence is shown for each cell population (10,000 cells per construct). FI, fluorescence intensity. Scalebar: 100 μm.

Three hours after transfection, cells were imaged every 30 min. The resulting time‐lapse (Figures 7a, S10, and Video S1) clearly shows that the green signal from mEos4Fast2 (and to a lesser extent mEos4Fast1) appears much earlier than that of mEos4b. Forty hours after transfection, mEos4Fast2 (and to a lesser extent, mEos4Fast1) continued to show a much brighter signal than mEos4b (Figure 7b), similarly to what we observed in living *E. coli* cells. This observation was confirmed by flow cytometry analysis (Figures 7c and S11).

We further questioned why mEos4Fast2 exhibits a higher fluorescence in cells and better resistance to PFA fixation, despite not having a higher molecular brightness measured on purified proteins (Table 1). Part of the explanation lies in the 5‐nm blue shift in its absorption spectrum as compared to mEos4b, with an absorption maximum at 499 nm better matching excitation by typical 488 nm light. This hypsochromic shift is also observed in the red form (Table 1 and Figure S12), well suited for excitation at 561 nm. Other explanations could involve better expression, solubility, or stability of mEos4Fast2 in cells, reduced cytotoxic effects, or better maturation yield.

To test these hypotheses, we conducted a series of control experiments. We initially quantified protein expression levels and maturation by extracting proteins from IPTG‐induced *E. coli* cultures (12 h post‐induction), followed by SDS‐PAGE analysis and UV–visible spectrophotometry (Figure S13).

The results revealed that mEos4Fast2 exhibited the highest maturation efficiency, despite similar protein expression levels for mEos4b and mEos4Fast2, and lower expression for mEos4Fast1. This higher maturation efficiency may account for the increased brightness observed for mEos4Fast2 both in prokaryotic and eukaryotic cells.

Subsequently, we assessed potential cytotoxic effects by measuring the 600‐nm turbidity of bacterial cultures before and 12 h after IPTG induction (Figure S14), but no significant differences in growth ratios among the three proteins could be noticed. Lastly, we evaluated thermal stability by incubating purified mEos4b, pcStar, mEosEM, mEos4Fast1, and mEos4Fast2 at temperatures ranging from 66 to 80°C and measuring residual fluorescence (Figure S15). mEos4Fast2 ranked second in thermal resistance, very close to mEosEM, suggesting that its structural robustness contributes to the stability of mEos4Fast2 in cells. These combined factors (efficient and faster maturation, protein stability and better resistance to fixation reagents) account for the consistently higher brightness of mEos4Fast2 observed in living and fixed cells.

### 2.7 Slow cyclization causes slow maturation of mEos4b

**FIGURE 8:**
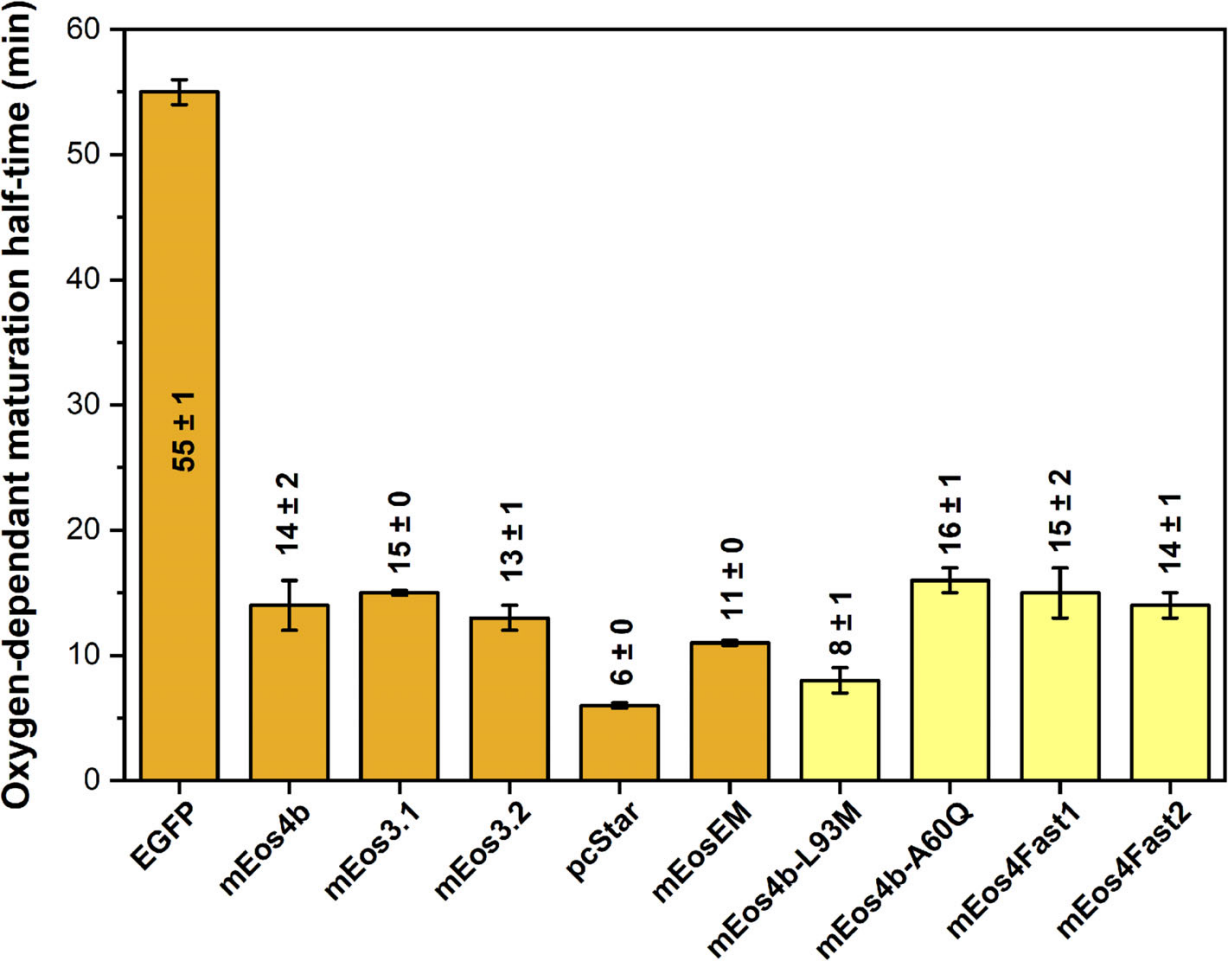
Comparison of half‐times for oxygen‐dependent chromophore forming step(s). Results are presented for EGFP (control), selected members of the mEos4b family (orange), mEos4Fast1, and mEos4Fast2, as well as some initial variants generated for this study (yellow). Error bars are standard deviations of time constants extracted from three separate measurements.

We further investigated whether pre‐oxidation or post‐oxidation events are responsible for the extremely slow maturation of mEos4b. Our aerobic maturation assay essentially measures the combination of all the processes involved in folding and maturation. We modified the assay to work under anaerobic conditions (see Section 5 and Figure S16, panels A and B), to ensure that the protein is produced and subsequently folds but, in the absence of oxygen, maturation of its chromophore stalls at the pre‐oxidation step(s). To confirm the effectiveness of the anaerobic conditions, we measured dissolved oxygen levels during the culture. Results showed that dissolved oxygen was completely consumed within 2 h and that the maximum level of reoxygenation at the start of the maturation measurement was 3% (Figure S16c). The fluorescence increase obtained upon subsequent exposure to oxygen then reflects the kinetics of the oxidation and (eventual) post‐oxidation events. Strikingly, under these conditions, the rise in fluorescence was fast, on the order of 10 min, for all tested mEos variants. This is in contrast with the results obtained from the aerobic maturation assay (Figure 8).

## 3 DISCUSSION

Our study tackles the significant challenge posed by the slow maturation of mEos4b, a widely used PCFP. Our results suggest that pre‐oxidation events are rate limiting for mEos4b and other slow‐maturing mEos derivatives, being considerably slower than post‐oxidation events. We aimed to identify or narrow down which steps of the pre‐oxidation events are actually rate limiting during mEos4b maturation: protein folding, cyclization, or dehydration for pathway A (Figure 1). Among these possibilities, protein folding can be excluded from this list because our NMR data showed that the slowly maturing species, observed in freshly prepared mEos4b samples, display only minor chemical shift differences compared to mature mEos4b (Figure 2). In the case of an unfolded, or only partially folded protein, much larger chemical shift differences would be expected. Among the two remaining possibilities, we speculate that cyclization is the rate‐limiting step. This hypothesis is supported by the fact that R91 has been shown to be a key residue to catalyze cyclization, while our findings indicate that mutations modulating the interaction of R91 with the chromophore are essential to accelerate maturation. However, it cannot be excluded that dehydration could also be rate limiting if pathway A is followed, as the enol form of the chromophore, promoted by R91, might be required for this step. This mechanistic insight into the early steps of chromophore maturation further supports the rationale behind the engineering of our fast‐maturing variants.

Our finding that the cyclization step may be rate‐limiting during mEos4b maturation is in stark contrast with previous observations in fast‐maturing FPs (Guerra et al., 2022; Nagai et al., 2002; Pouwels et al., 2008), where cyclization occurs rapidly, and the rate‐limiting steps involve oxygen‐dependent processes. This difference is confirmed by our EGFP maturation data measured under anaerobic conditions (Figure 4), revealing a much slower oxidation step compared to all mEos‐derived variants. This suggests that the free energy barriers associated with the various maturation steps strongly differ between hydrozoan and anthozoan proteins. Given the relatively low sequence identity (~30%) between FPs from the two families, this difference may not be so surprising. However, further investigations will be necessary to explore this issue in greater detail.

This slow maturation compromises effective labeling efficiency and may hinder real‐time tracking of dynamic processes. We developed a strategy to generate fast‐maturing variants of mEos4b, which are functional both in bacteria and mammalian cells. Importantly, this accelerated maturation does not compromise the biochemical and photophysical properties of the protein. The variants retain the favorable properties of the parent mEos4b at both the ensemble and single‐molecule levels, ensuring reliable performance in advanced fluorescence microscopy.

The rational design approach taken herein was tailored to the specific chromophore environment of the mEos family. The mutations introduced near residue R91 (key catalyst of the chromophore cyclization) were highly effective at accelerating maturation in this protein family. While this strategy shows the potential of targeted modifications based on the local chemical environment of the chromophore, it is not directly transferable to other FPs with alternative structural contexts. We attempted to introduce the same mutations into unrelated FPs, including the photoconvertible Dendra2, the reversibly switchable Dronpa, and the red‐emitting FusionRed. These attempts were not successful: either maturation was still slow or the proteins did not fold properly. These results point out that any successful attempt to accelerate maturation by rational design must take into account the atomic environment of the chromophore and that strategies should be customized on a case‐by‐case basis for each FP scaffold.

Our work provides a comprehensive comparison of the maturation rates among various mEos‐derived PCFPs, revealing significant differences between them. Although absolute values may be influenced by the methodology used, our measurements offer a reliable comparison. When maturation speed is critical, we recommend using mEosEM, pcStar or one of our fast‐maturing mEos4b variants for their experiments. All four PCFPs exhibit relatively similar properties. Utilizing these fast‐maturing PCFPs should enable real‐time visualization of dynamic events at higher resolution, which is essential for studying proteins with short half‐lives, or observing protein synthesis, modification, and degradation without the delays associated with slower‐maturing chromophores.

Significant acceleration of mEos4b maturation was achieved by modifying the immediate environment of R91, a residue known to play a key role in catalyzing maturation. In the absence of a structure for the pre‐cyclized chromophore, the exact mechanism by which the introduced mutations alter the interaction of R91 with the carbonyl group of G64 (the third amino acid of the chromophore) remains to be clarified. We hypothesize that a slight repositioning of R91 and/or changes in the charge distribution at the guanidinium group could favor the cyclization step, and/or possibly the dehydration step during maturation. High‐level theoretical calculations may provide additional insights into the exact mechanisms involved.

In the absence of high‐resolution structures for mEos4Fast1 and mEos4Fast2, our mechanistic interpretation remains provisional. We can however note that the crystal structure of mEos4b‐L93M, AlphaFold‐3/MD models of the fast variants, and their comparative maturation kinetics offer a coherent framework for the synergy of positions 59‐60‐93 and the additional rate boost conferred by distal surface substitutions.

First, the L93M mutation enlarges a hydrophobic pocket adjacent to the chromophore and stabilizes a single ordered water molecule (W1) that bridges T59 and R91, subtly repositioning the catalytic arginine. In the A60Q/L93M context, modeling suggests that the amide of Q60 can replace W1 and form a persistent bidentate hydrogen bond with R91 (an interaction that cannot be made when A60Q is introduced on its own, probably because the native pocket is too narrow and potentially lacks W1). This pocket pre‐organization rationalizes why A60Q alone only modestly shortens maturation (~9‐fold, 209 min), whereas the double mutant mEos4Fast1 reaches 64 min. By contrast, in the L93M crystal structure, the native hydroxyl of T59 already forms hydrogen bonds to R91 and to the ordered water molecule W1. Replacing threonine with glutamine therefore lengthens the side chain and introduces an amide that can engage R91 directly, potentially in a bidentate fashion, without requiring the more extensive pocket reorganization inferred for A60Q. Consistent with this idea, the single mutant mEos4b‐T59Q matures in 131 min, and combining T59Q with L93M produces mEos4Fast2, the fastest variant so far (27 min). These observations suggest a synergistic contribution of positions 59 and 93, with only limited additional pocket adjustment needed for T59Q compared with A60Q.

Second, mEosEM carries two additional surface substitutions (D28E on β‐strand 1 and N166G in the β8–β9 loop) that lie outside the β‐barrel yet correlate with a further three‐fold acceleration relative to mEos4b‐L93M. Although their side‐chains do not point towards the chromophore, the Asp to Glu change conserves charge while extending the hydrogen‐bond network along the barrel axis, whereas the Asn to Gly change increases loop flexibility and may transiently widen solvent channels. Similar distal tuning is exemplified by superfolder GFP, where several remote mutations at the surface of the protein enhance its properties (Pedelacq et al., 2006).

Collectively, these observations support a model in which maturation speed in the EosFP scaffold is governed by an interplay between (i) local pocket chemistry that controls the H‐bond network linking R91 to the pre‐cyclized chromophore and (ii) distant surface mutations that modulate folding pathways and solvent permeability.

In PALM, sptPALM, or MINFLUX, fast‐maturing mEos variants may be indispensable for some experiments, both in fixed or live cells. First, a swift fluorescence onset is essential to rapidly capture at the nanoscale molecular patterns triggered by, for example, drug administration. Second, fast and efficient maturation can be particularly crucial, depending on target‐protein turnover, to maximize effective labeling efficiency in counting experiments aimed at measuring protein complex stoichiometries. Third, achieving sufficient maturation efficiency is central to the reconstruction of truly high‐resolution nanoscopy images and for providing a faithful representation of the spatial distribution of low‐abundance proteins.

As compared to mEos4Fast1, mEos4Fast2 matures even faster and has a lower pKa, leading to higher overall brightness in live cells. Furthermore, mEos4Fast2 displays strong resistance to chemical fixation. Thus, this novel mEos variant is expected to perform exceptionally well in live‐ or fixed‐cell applications, making it a marker of choice for pulse‐chase ensemble or single‐molecule‐based super‐resolution imaging, including correlative studies. mEos4Fast1, on the other hand, appears better suited for CLEM or single‐particle tracking experiments, for which a reduced blinking propensity is crucial for accurate tracking. Interestingly, our results also reveal that thermal stability, PFA resistance, and OsO_4_ resistance are not directly correlated. Taken together, these findings underscore the complementarity of mEos4Fast1 and mEos4Fast2, which should be selected according to the specific constraints and imaging requirements of each experimental context.

## 4 CONCLUSIONS

The development of fast‐maturing PCFPs is needed to enhance the quality of data obtained from advanced fluorescence microscopy techniques. In addition to mEosEM and pcStar, our new fast‐maturating mutants mEos4Fast1 and mEos4Fast2 are expected to be particularly beneficial for PALM and MINFLUX super‐resolution microscopy, addressing the limitations of the very slow‐maturing mEos2, mEos3.1, mEos3.2, and mEos4b variants and providing a reliable tool for capturing transient and dynamic cellular events. In several aspects, the variants we propose perform comparably to more recent members of the mEos family (sometimes better, sometimes less so), highlighting their potential as practical alternatives in specific imaging contexts.

Our study is the first to propose a rational design approach for engineering fast‐maturing variants of a photoconvertible fluorescent protein. It provides new mechanistic insights into the maturation mechanism of anthozoan FPs. However, further research is needed to fully understand the precise role of the key mutations introduced around R91 in accelerating maturation. Additionally, evaluating the performance of our fast‐maturing mEos4b variants across a diverse range of experimental conditions and biological systems will be essential for expanding their applicability. Lastly, applying our strategy to other slow‐maturing FPs, including other PCFPs, could advance various fields of biological and biomedical research, enabling faster and more precise cell imaging.

## 5 METHODS

### 5.1 Cloning

All the proteins studied were cloned in a modified pET28a vector carrying the gene for kanamycin resistance, with the exception of mEos2, mEos3.1, and mEos3.2, which were previously cloned in the pRSET‐A vector carrying the gene for ampicillin resistance. The DNA template of mEos2 was ordered at Addgene (plasmid # 20341) (McKinney et al., 2009), as were those of mEos3.2 and EGFP (plasmid # 58470) (Belin et al., 2014). Synthetic constructs of mEos4b, mEos4b‐V69T, pcStar, mEosEM, mStayGold, and mNeonGreen were ordered at Genecust. All the other proteins described in this study were obtained by in‐house mutations.

### 5.2 Mutagenesis

Single point mutants were prepared in‐house using the Q5® Site‐directed mutagenesis kit (New England Biolabs) and primers whose list can be found in Table S2.

### 5.3 Mammalian bicistronic constructs

For enhanced mammalian expression, the mEos4b sequence was optimized by incorporating a Kozak sequence and N‐terminal (N‐ter) and C‐terminal (C‐ter) sequences from EGFP, a widely adopted strategy (Shaner et al., 2004). The two N‐terminal amino acids of mEos4b (MS) were substituted via PCR amplification with the 11‐residue GFP‐type N‐terminus sequence (MVSKGEEDNMA). Concurrently, the Kozak sequence (GCCACCATGG) was inserted between the *Nhe*I site and overlapping the initiation codon of the mEos4b coding sequence. Similarly, the C‐terminal sequence of mEos4b (DNARR) was replaced with the 7‐residue GFP‐type C‐terminus sequence (GMDELYK) in a symmetrical manner. A 24‐residue linker (GSGEGRGSLLTCGDVEENPGPRSL) was also inserted immediately after the mEos4b sequence and overlapping the *Age*I restriction site. This linker codes for the underlined 18‐residue 2A peptide from the *Thosea asigna* virus capsid protein, which is self‐cleaving between the last two amino acids (proline and glycine) and ensures an equimolar translation of the two fluorescent proteins. This sequence was cloned between the *Nhe*I and *Age*I restriction sites in the MCS, upstream of mCherry in the mammalian plasmid pmCherry_N1 (Clontech) and mutations T59Q, A60Q, and L93M were then added to mEos4b, resulting in the three bicistronic constructs mEos4b‐2a‐mCherry_N1, mEos4Fast1‐2a‐mCherry_N1, and mEos4Fast2‐2a‐mCherry_N1.

### 5.4 Protein production and purification

*Escherichia coli* BL21(DE3) cells were transformed with a plasmid carrying the target protein. Cells were adapted from rich LB medium to M9 minimal medium isotopically enriched with ^15^N NH_4_Cl (1 g/L) and ^13^C‐glucose (2 g/L) in two steps over 24 h. For proteins prepared for X‐ray crystallography, the adaptation from LB to M9 minimal media was not necessary; therefore, all steps of cell growth were performed in LB medium. Cells were grown at 37°C until the turbidity measured at 600 nm reached >0.5. Protein over‐expression was induced by adding 1 mM IPTG (isopropyl β‐D‐thiogalactopyranoside). After overnight induction at 20°C, cells were harvested and sonicated in 20 mM HEPES buffer at pH 7.5 supplemented with 150 mM NaCl and a cOmplete™ EDTA‐Free (Roche) tablet. The lysate was centrifuged at 46,000 × *g* for 40 min. The supernatant was collected and passed through a Ni‐NTA (Qiagen) column pre‐equilibrated with the buffer mentioned before. Protein elution was performed with 500 mM imidazole. Size exclusion chromatography was performed with a S75 (Cytiva) column equilibrated with 20 mM HEPES pH 7.5, 150 mM NaCl buffer. Elution fractions containing the protein were dialyzed against the 20 mM HEPES buffer at pH 7.5. Purified samples were stored at −80°C until needed.

### 5.5 NMR experiments

NMR samples were prepared in 5 mm Shigemi NMR tubes, containing 100–200 μM of protein in 300 μL of buffer solution with 5% (v/v) D_2_O, filled. NMR experiments were performed on a Bruker Avance III‐HD spectrometer (850 MHz) equipped with a triple‐resonance cryo‐probe and pulsed z‐field gradients. Unless otherwise specified, all measurements were performed at 35°C. Data were processed using TopSpin v3.5 (Bruker BioSpin). Data analysis was performed with the CCPNMR v2 software. 2D ^1^H‐^15^N amide backbone correlation spectra were recorded using a BEST‐TROSY pulse sequence (Favier & Brutscher, 2011) with the ^15^N carrier centered at 120 ppm, and the ^1^H band selective pulses covering a band width of 8.7 ± 2.5 ppm.

### 5.6 Fluorescence‐based maturation assays

Maturation assay performed in aerobic condition consisted of a similar protocol for protein production described above, consisting of transformation, cell growth in LB medium and induction by IPTG at 37°C for 1–2 h. A cocktail of antibiotics containing chloramphenicol (final concentration 0.17 mg/mL) and tetracycline (final concentration 0.05 mg/mL) was then added to the media containing the cells to stop fresh protein production. 100 μL of the solution was transferred in well(s) of a 96‐well plate. Fluorescence signal was measured in a BioTek microplate reader. Fluorescence signal was excited at 470 nm, and the emission data were integrated between 495 and 535 nm. The signal corresponding to LB medium only were subtracted from each dataset. The data were then baseline corrected for any turbidity variations measured at 600 nm during the measurement process. This ensured no data artifacts were introduced due to delay in antibiotic functioning resulting in residual cell division and cell death. Homemade python scripts were used to perform data analyses. The data were plotted as a function of time to follow the maturation process. Most datasets fit to a monoexponential *y* = *A*·*e* ^−*t*/*τ*^ + *B* equation, *τ* being the apparent maturation time. Fluorescence maturation curve of mEos4b and certain other slow maturing variants such as mEos2, mEos3.2, mEos4b‐I157V, and even some faster maturing variants such as mEos4b‐T59Q were accompanied by a slow phase. In such cases, the data were fit to the point until which a monoexponential function could fit well. The maturation time reported in this study is the monoexponential time constant obtained from the fitting. All experiments were triplicated.

In order to perform the maturation assay in anaerobic conditions, the following adjustments to the assay were made. The cells were grown overnight in the presence of oxygen in LB medium at 37°C. Following this, a subculture was done by diluting the cells 100 times in Terrific Broth medium supplemented with 50 mM MOPS‐KOH pH 7.1, 0.5% D‐glucose, and the necessary antibiotic (Kanamycin/Ampicillin). Once the bacterial turbidity reached 0.5–0.6, cultures were transferred into glass containers and sealed with caps containing a rubber septum. After a 20‐min incubation period at 37°C (to ensure all the residual oxygen is consumed by the cells), the culture was supplemented with ammonium ferric citrate (40 μM), L‐cysteine (400 μM), sodium fumarate (25 mM), vitamin cocktail mix and IPTG by means of injection through the rubber septum. Following a 2‐h induction at 37°C, the antibiotic cocktail containing chloramphenicol (final concentration 0.17 mg/mL) and tetracycline (final concentration 0.05 mg/mL) was added to the culture. The oxygen level was measured during the bacterial growth thanks to a Pico‐O2 optical oxygen meter (Pyroscience GmbH) and at this step, there was already almost no measurable oxygen in the cultures. The culture bottles were transferred to 20°C overnight. The following morning, the bottles were opened and 100 μL of culture from each bottle was transferred to 96‐well plates for fluorescence measurements. The time differences between opening each bottle and the start of the measurement (typically 2 min) were taken into account during data analyses and correspond to a maximum of 3% of oxygen. A graphical illustration of the assays as well as the measurement of oxygen levels during the culture is shown in Figure S16.

### 5.7 Ensemble spectroscopic properties and pKa

#### 5.7.1 Extinction coefficient

The molar extinction coefficient of the green form of all PCFPs reported in Table 1 was determined using the Ward method (Ward, 1981). The extinction coefficient of the free GFP‐like chromophore is known to be 44,000M^−1^ cm^−1^ at 447 nm. Comparing the absorption spectra of the native proteins at pH 7.5 and fully denatured proteins with still intact chromophore after gradual addition of 1M NaOH, the molar extinction coefficient at their absorption maxima was calculated. The molar extinction coefficient of the red form of the PCFPs was obtained by comparing the decrease in absorption in the green form and increase in absorption of the red form (Subach et al., 2011). PCFPs were photoconverted in a cuvette (with a starting green‐form OD 0.5–0.7) with a 405 nm laser of power density 250 mW/cm^2^.

#### 5.7.2 Quantum yield

The quantum yield values were measured by comparing them with known standards, fluorescein in 0.1 M NaOH solution for the green forms and rhodamine 6G in ethanol solution for the red forms, considering quantum yields equal to 0.92 and 0.95 for fluorescein and rhodamine 6G, respectively (Magde et al., 2002). The fluorescence spectra of the green and red forms were obtained using an excitation laser of 473 and 532 nm, respectively.

#### 5.7.3 pKa

Solution of the green and red forms of the PCFPs was prepared at different pH values ranging from 4.2 to 9.0. From the absorption spectra of the protein solutions, the amplitude of the absorption peaks corresponding to either the neutral or the anionic form was plotted as a function of pH. pKa values were obtained by fitting the following equation.

$$Y=A_{\min}+\left(A_{\max}-A_{\min}\right)/1+10^{\left(\mathrm{pKa}-\mathrm{pH}\right)}$$

### 5.8 Crystallization and crystallographic characterization

Purified mEos4b‐L93M was concentrated by ultrafiltration and equilibrated in buffer solution (50 mM HEPES pH 7.5). Crystals were obtained by the hanging‐drop vapor diffusion method at 20°C. Briefly, the protein (15 mg/mL) and precipitant solution (0.1M HEPES pH 8.5 and 32% PEG 1000) were mixed 1:1, yielding 2‐μL drops that were placed over a 1 mL well containing the precipitant solution. Needle‐shaped crystals appeared within 7 days. Prior to data collection, crystals of mEos4b‐L93M were cryoprotected by a short soak in the mother liquor supplemented with 20% glycerol, followed by flash‐cooling in liquid nitrogen. X‐ray diffraction data sets were collected at 100 K at the European Synchrotron Radiation Facility (ESRF) on the beamline ID30‐A3/MASSIF‐3 (von Stetten et al., 2020), equipped with an Eiger1 X 4M detector and a wavelength set to 0.9677 Å. Data were processed via the autoPROC pipeline (Vonrhein et al., 2011) using XDS (Kabsch, 2010) and POINTLESS (Evans, 2011) to integrate and merge data in the P2_1_2_1_2_1_ space group and AIMLESS (Evans & Murshudov, 2013) for scaling. The structure was phased by the molecular replacement method using as a starting model the X‐ray structure of mEos4b (PDB ID: 6GOY) and the program Phaser.MRage (Bunkoczi et al., 2013). Model building was performed with Coot (Emsley et al., 2010). Refinement and map calculations were performed using Phenix (Liebschner et al., 2019) at 1.86 Å resolution. Data collection and refinement statistics are provided in Table S3. Figures were produced using PyMOL (Schrodinger, 2015).

### 5.9 Apparent brightness in bacteria

*E. coli* cells were transformed with plasmids containing the coding sequence of either mEos4b, mEos4Fast1, or mEos4Fast2 under the control of the T7 promoter. Cultures were let to grow at 37°C until reaching a turbidity of 0.5 at 600 nm. Cultures were then induced with 10 mM IPTG, immediately plated on an LB‐agar gelose, and let at room temperature in the dark. From 4 h after plating, photographs were taken regularly under a blue light transilluminator, over a period of 24 h.

### 5.10 Transfection

We chose U2OS cells for their high transfection efficiency and their flat morphology that make them very often used for cell imaging. U2OS cells were seeded at a density of 0.4 × 10^6^ cells per well in six‐well plates containing growth medium (phenol red‐free DMEM supplemented with 10% FBS). The following day, the cells were transfected with bicistronic constructs, including mEos4b‐2A‐mCherry, mEos4Fast1‐2A‐mCherry or mEos4Fast2‐2A‐mCherry, using the Fugene HD reagent (Promega) as per the manufacturer's instructions. The transfected cells were then incubated at 37°C with 5% CO_2_ for up to 40 h, depending on the subsequent analysis method.

### 5.11 Apparent brightness in human cells

Fluorescence microscopy experiments were conducted using U2OS cells transfected with either the mEos4b‐2a‐mCherry, the mEos4Fast1‐2a‐mCherry or the mEos4Fast2‐2a‐mCherry construct. Following transfection, cells were cultured in FluoroBrite™ DMEM (Gibco) medium to minimize autofluorescence, supplemented with 10% FBS, GlutaMAX (Gibco) and MEM NEAA (Gibco). Cells were then observed under a spinning disk microscope, Olympus IX81, equipped with a Yokogawa CSU‐X1 confocal head, a motorized stage and an incubation chamber (Okolab), which provides a 5% CO_2_ atmosphere at 37°C to maintain a stable pH throughout the live cell imaging. Time‐lapse acquisition commenced as early as 4 h post‐transfection, with the time interval between frames set to 30 min. The sample was excited with a 488‐nm laser at 0.3 mW (green channel, Eos variants) and a 561‐nm laser at 0.13 mW (red channel, mCherry). Laser powers were measured at the sample level (Ilas2 laser bench, GATACA systems). The fluorescence signal was collected with a 10× 0.25 NA air‐objective and filtered with individual band‐pass filters Semrock FF02‐520/28 and Chroma ET600/50m, for green and red channels respectively, before being detected by an EMCCD camera (Andor iXon Ultra). Fluorescence kinetics were monitored within cells for a period of 44 h. The green fluorescence signal was normalized to the red signal originating from mCherry, allowing for accurate quantification and comparison of fluorescence levels between experimental conditions.

### 5.12 Fixation and osmium resistance assay

Fluorescence conservation ability of the PCFPs in presence of fixating agents PFA and GA was tested based on methods demonstrated before (Osuga et al., 2021) or in presence of OsO_4_. In brief, purified proteins were diluted in PBS (pH 7.2) and incubated for 30 min at 37°C either without any fixative agents or with (A) 4% PFA and (B) 4% PFA + 0.2% GA or incubated for 10 min with either 0.5%, 1.0%, or 1.5% OsO_4_. Following which fluorescence intensity was measured in a BioTek microplate reader. For fixating reagents, measurements were also performed on transfected U2OS cells. Twenty‐four hours after transfection, the fluorescence of the same harvested cell sample was analyzed by flow cytometry, before fixation (live cells), after 30 min of treatment with 4% PFA, or with 4% PFA + 0.2% GA. Fixation resistance was calculated based on the ratio of fluorescence intensity of samples incubated with fixatives to the corresponding controls (without fixatives). The data is presented in Figure S8.

### 5.13 Flow cytometry

Following transfection, the cells were maintained in culture for 8, 16, 24, or 40 h at 37°C with 5% CO_2_. After the designated incubation period, the cells were harvested using 0.25% trypsin/EDTA. The trypsinization process was halted by adding medium containing 10% FBS. The cells were then pelleted by centrifugation at 300 × *g* for 5 min, washed, and resuspended in PBS for flow cytometry analysis. Flow cytometry was performed using a MACSQuant VYB Cytometer (Miltenyi Biotec), with a minimum of 10,000 events collected within the analysis gate. mCherry and green state mEos4 fluorescence were acquired simultaneously. Data were analyzed using MACSQuantify software.

### 5.14 Structure prediction and refinement of mEos4b A60Q/L93M mutants

We employed AlphaFold 3 (Abramson et al., 2024), a cutting‐edge protein structure prediction tool (available at https://golgi.sandbox.google.com), to simulate the 3D structure of mEos4b carrying the A60Q and L93M mutations, starting from the amino acid sequence. Concurrently, the dynamics and minimization (phenix.dynamics) module of Phenix version 1.20.1‐4487 (Liebschner et al., 2019) was employed, using the crystallographic structure of mEos4b‐L93M as a starting model. Prior to this, the A60Q mutation had been introduced and water molecule W1 removed, using Coot v. 0.9.8.93 (Emsley et al., 2010).

### 5.15 Chromophore and protein yields for mEos4b, mEos4Fast1, and mEos4Fast2

*E. coli* beta‐10 (NEB) colonies expressing either mEos4b, mEos4Fast1, or mEos4Fast2 were inoculated in LB medium supplemented with kanamycin. Cultures were grown at 37°C until reaching a turbidity of 0.5 at 600 nm. Protein expression was induced in 4 mL culture with 10 mM IPTG, followed by overnight incubation at 37°C. Cells were harvested, and proteins were extracted using BugBuster Protein Extraction Reagent (MilliporeSigma, Cat. No. 70584‐3) according to the manufacturer's protocol. The extracts were analyzed by SDS‐PAGE and UV–visible spectrophotometry to determine protein concentration (based on the molar extinction coefficients reported in Table 1) and maturation.

### 5.16 Single‐molecule fluorescence measurements

Purified proteins (mEos4b, mEos4Fast1, and mEos4Fast2) were immobilized in a polyacrylamide gel (pH 8.0) and single‐molecule imaging was performed using laser excitations at 561 nm (500 W/cm^2^, 70 ms exposure time) and 405 nm (1 W/cm^2^, 8.2 ms exposure time). Fluorescence was collected by a 100× 1.49 NA oil‐immersion objective (Olympus), passed through a dichroic mirror (FF580‐FDi01‐25x36, Semrock), and filtered using an emission filter (FF01‐630/92‐25, Semrock). An additional emission filter (FF01‐612/69, Semrock) was mounted in a filter wheel in front of the EMCCD camera (Evolve 512, Photometrics) with a pixel size of 128 nm/pixel.

Molecules were localized using the ThunderSTORM ImageJ plugin (Ovesny et al., 2014) and localizations were clustered using an in‐house MATLAB routine to reconstruct fluorescence time traces belonging to single molecules (Wulffele et al., 2022). From these fluorescence time traces, off‐times, on‐times, bleaching‐times, number of blinks, and photon budget were extracted. All proteins behave similarly under these conditions.

### 5.17 Oligomeric state determination

The oligomeric state of mEos4b, mEos4Fast1, and mEos4Fast2 was evaluated using Size Exclusion Chromatography coupled with Multi‐Angle Light Scattering (SEC‐MALS). Experiments were performed using an OMNISEC RESOLVE system (Malvern Panalytical) at the Protein Analysis On Line (PAOL) platform (ISBG—Integrated Structural Biology Grenoble). The setup included a pump, a temperature‐controlled autosampler, and a column oven, connected to an OMNISEC REVEAL module comprising a UV–visible absorbance detector, a differential refractive index detector, a multi‐angle light scattering detector (RALS at 90° and LALS at 7°), and a differential viscometer. A 50 μL volume of each protein sample (at 1 mg/mL) was injected onto a Superdex 75 10/300 GL column (Cytiva) pre‐equilibrated at 25°C with an elution buffer composed of 20 mM HEPES pH 7.5 and 150 mM NaCl. The separation was performed at a flow rate of 0.5 mL/min. Data were acquired and analyzed using the OMNISEC v11.40 software.

## AUTHOR CONTRIBUTIONS

**Arijit Maity:** Conceptualization; methodology; data curation; investigation. **Oleksandr Glushonkov:** Investigation; data curation. **Isabel Ayala:** Investigation. **Pascale Tacnet:** Investigation; data curation. **Jip Wulffelé:** Data curation. **Philippe Frachet:** Investigation. **Bernhard Brutscher:** Supervision; funding acquisition. **Dominique Bourgeois:** Funding acquisition; writing – review and editing; supervision. **Virgile Adam:** Writing – review and editing; writing – original draft; conceptualization; supervision; data curation; methodology; project administration.

## CONFLICT OF INTEREST STATEMENT

There are no conflicts to declare.

## Supporting information


**Appendix S1:** Supporting information.

## Data Availability

The data that support the findings of this study are available from the corresponding author upon reasonable request.

## References

[pro70234-bib-0001] Abramson J , Adler J , Dunger J , Evans R , Green T , Pritzel A , et al. Accurate structure prediction of biomolecular interactions with AlphaFold 3. Nature. 2024;630:493–500. 38718835 10.1038/s41586-024-07487-w PMC11168924

[pro70234-bib-0002] Ando R , Shimozono S , Ago H , Takagi M , Sugiyama M , Kurokawa H , et al. StayGold variants for molecular fusion and membrane‐targeting applications. Nat Methods. 2024;21:648–656. 38036853 10.1038/s41592-023-02085-6 PMC11009113

[pro70234-bib-0003] Auhim HS , Grigorenko BL , Harris TK , Aksakal OE , Polyakov IV , Berry C , et al. Stalling chromophore synthesis of the fluorescent protein Venus reveals the molecular basis of the final oxidation step. Chem Sci. 2021;12:7735–7745. 34168826 10.1039/d0sc06693a PMC8188506

[pro70234-bib-0004] Balleza E , Kim JM , Cluzel P . Systematic characterization of maturation time of fluorescent proteins in living cells. Nat Methods. 2018;15:47–51. 29320486 10.1038/nmeth.4509 PMC5765880

[pro70234-bib-0005] Balzarotti F , Eilers Y , Gwosch KC , Gynna AH , Westphal V , Stefani FD , et al. Nanometer resolution imaging and tracking of fluorescent molecules with minimal photon fluxes. Science. 2017;355:606–612. 28008086 10.1126/science.aak9913

[pro70234-bib-0006] Barondeau DP , Putnam CD , Kassmann CJ , Tainer JA , Getzoff ED . Mechanism and energetics of green fluorescent protein chromophore synthesis revealed by trapped intermediate structures. Proc Natl Acad Sci U S A. 2003;100:12111–12116. 14523232 10.1073/pnas.2133463100 PMC218721

[pro70234-bib-0007] Barondeau DP , Tainer JA , Getzoff ED . Structural evidence for an enolate intermediate in GFP fluorophore biosynthesis. J Am Chem Soc. 2006;128:3166–3168. 16522096 10.1021/ja0552693

[pro70234-bib-0008] Belin BJ , Goins LM , Mullins RD . Comparative analysis of tools for live cell imaging of actin network architecture. Bioarchitecture. 2014;4:189–202. 26317264 10.1080/19490992.2014.1047714 PMC4914014

[pro70234-bib-0009] Berardozzi R , Adam V , Martins A , Bourgeois D . Arginine 66 controls dark‐state formation in green‐to‐red photoconvertible fluorescent proteins. J Am Chem Soc. 2016;138:558–565. 26675944 10.1021/jacs.5b09923

[pro70234-bib-0010] Bevis BJ , Glick BS . Rapidly maturing variants of the Discosoma red fluorescent protein (DsRed). Nat Biotechnol. 2002;20:83–87. 11753367 10.1038/nbt0102-83

[pro70234-bib-0011] Bunkoczi G , Echols N , McCoy AJ , Oeffner RD , Adams PD , Read RJ . Phaser.MRage: automated molecular replacement. Acta Crystallogr D Biol Crystallogr. 2013;69:2276–2286. 24189240 10.1107/S0907444913022750 PMC3817702

[pro70234-bib-0012] Craggs TD . Green fluorescent protein: structure, folding and chromophore maturation. Chem Soc Rev. 2009;38:2865–2875. 19771333 10.1039/b903641p

[pro70234-bib-0013] Cubitt AB , Heim R , Adams SR , Boyd AE , Gross LA , Tsien RY . Understanding, improving and using green fluorescent proteins. Trends Biochem Sci. 1995;20:448–455. 8578587 10.1016/s0968-0004(00)89099-4

[pro70234-bib-0014] De Zitter E , Thedie D , Monkemoller V , Hugelier S , Beaudouin J , Adam V , et al. Mechanistic investigation of mEos4b reveals a strategy to reduce track interruptions in sptPALM. Nat Methods. 2019;16:707–710. 31285624 10.1038/s41592-019-0462-3

[pro70234-bib-0015] Emsley P , Lohkamp B , Scott WG , Cowtan K . Features and development of coot. Acta Crystallogr D Biol Crystallogr. 2010;66:486–501. 20383002 10.1107/S0907444910007493 PMC2852313

[pro70234-bib-0016] Evans PR . An introduction to data reduction: space‐group determination, scaling and intensity statistics. Acta Crystallogr D Biol Crystallogr. 2011;67:282–292. 21460446 10.1107/S090744491003982X PMC3069743

[pro70234-bib-0017] Evans PR , Murshudov GN . How good are my data and what is the resolution? Acta Crystallogr D Biol Crystallogr. 2013;69:1204–1214. 23793146 10.1107/S0907444913000061 PMC3689523

[pro70234-bib-0018] Favier A , Brutscher B . Recovering lost magnetization: polarization enhancement in biomolecular NMR. J Biomol NMR. 2011;49:9–15. 21190063 10.1007/s10858-010-9461-5

[pro70234-bib-0019] Fu Z , Peng D , Zhang M , Xue F , Zhang R , He W , et al. mEosEM withstands osmium staining and Epon embedding for super‐resolution CLEM. Nat Methods. 2020;17:55–58. 31611693 10.1038/s41592-019-0613-6

[pro70234-bib-0020] Gadella TWJ Jr , van Weeren L , Stouthamer J , Hink MA , Wolters AHG , Giepmans BNG , et al. mScarlet3: a brilliant and fast‐maturing red fluorescent protein. Nat Methods. 2023;20:541–545. 36973546 10.1038/s41592-023-01809-y

[pro70234-bib-0021] Ganini D , Leinisch F , Kumar A , Jiang J , Tokar EJ , Malone CC , et al. Fluorescent proteins such as eGFP lead to catalytic oxidative stress in cells. Redox Biol. 2017;12:462–468. 28334681 10.1016/j.redox.2017.03.002 PMC5362137

[pro70234-bib-0022] Grigorenko BL , Krylov AI , Nemukhin AV . Molecular modeling clarifies the mechanism of chromophore maturation in the green fluorescent protein. J Am Chem Soc. 2017;139:10239–10249. 28675933 10.1021/jacs.7b00676

[pro70234-bib-0023] Guerra P , Vuillemenot LA , Rae B , Ladyhina V , Milias‐Argeitis A . Systematic in vivo characterization of fluorescent protein maturation in budding yeast. ACS Synth Biol. 2022;11:1129–1141. 35180343 10.1021/acssynbio.1c00387 PMC8938947

[pro70234-bib-0024] Heim R , Prasher DC , Tsien RY . Wavelength mutations and posttranslational autoxidation of green fluorescent protein. Proc Natl Acad Sci U S A. 1994;91:12501–12504. 7809066 10.1073/pnas.91.26.12501 PMC45466

[pro70234-bib-0025] Kabsch W . XDS. Acta Crystallogr D Biol Crystallogr. 2010;66:125–132. 20124692 10.1107/S0907444909047337 PMC2815665

[pro70234-bib-0026] Kim H , Grunkemeyer TJ , Modi C , Chen L , Fromme R , Matz MV , et al. Acid‐base catalysis and crystal structures of a least evolved ancestral GFP‐like protein undergoing green‐to‐red photoconversion. Biochemistry. 2013;52:8048–8059. 24134825 10.1021/bi401000e

[pro70234-bib-0027] Lambert TJ . Using FPbase: the fluorescent protein database. Methods Mol Biol. 2023;2564:1–45. 36107335 10.1007/978-1-0716-2667-2_1

[pro70234-bib-0028] Lemay NP , Morgan AL , Archer EJ , Dickson LA , Megley CM , Zimmer M . The role of the tight‐turn, broken hydrogen bonding, Glu222 and Arg96 in the post‐translational Green fluorescent protein chromophore formation. Chem Phys. 2008;348:152–160. 19079566 10.1016/j.chemphys.2008.02.055 PMC2597819

[pro70234-bib-0029] Liebschner D , Afonine PV , Baker ML , Bunkoczi G , Chen VB , Croll TI , et al. Macromolecular structure determination using X‐rays, neutrons and electrons: recent developments in Phenix. Acta Crystallogr D Struct Biol. 2019;75:861–877. 31588918 10.1107/S2059798319011471 PMC6778852

[pro70234-bib-0030] Liu B , Mavrova SN , van den Berg J , Kristensen SK , Mantovanelli L , Veenhoff LM , et al. Influence of fluorescent protein maturation on FRET measurements in living cells. ACS Sens. 2018;3:1735–1742. 30168711 10.1021/acssensors.8b00473 PMC6167724

[pro70234-bib-0031] Ma Y , Sun Q , Zhang H , Peng L , Yu JG , Smith SC . The mechanism of cyclization in chromophore maturation of green fluorescent protein: a theoretical study. J Phys Chem B. 2010;114:9698–9705. 20593847 10.1021/jp1039817

[pro70234-bib-0032] Ma Y , Sun Q , Smith SC . The mechanism of oxidation in chromophore maturation of wild‐type green fluorescent protein: a theoretical study. Phys Chem Chem Phys. 2017;19:12942–12952. 28480935 10.1039/c6cp07983k

[pro70234-bib-0033] Ma YY , Yu JG , Sun Q , Li Z , Smith SC . The mechanism of dehydration in chromophore maturation of wild‐type green fluorescent protein: a theoretical study. Chem Phys Lett. 2015;631:42–46.

[pro70234-bib-0034] Magde D , Wong R , Seybold PG . Fluorescence quantum yields and their relation to lifetimes of rhodamine 6G and fluorescein in nine solvents: improved absolute standards for quantum yields. Photochem Photobiol. 2002;75:327–334. 12003120 10.1562/0031-8655(2002)075<0327:fqyatr>2.0.co;2

[pro70234-bib-0035] Maity A , Wulffele J , Ayala I , Favier A , Adam V , Bourgeois D , et al. Structural heterogeneity in a phototransformable fluorescent protein impacts its photochemical properties. Adv Sci (Weinh). 2024;11:e2306272. 38146132 10.1002/advs.202306272 PMC10933604

[pro70234-bib-0036] Marathe P , Sm H , Nair D , Bhattacharyya D . mEosBrite are bright variants of mEos3.2 developed by semirational protein engineering. J Fluoresc. 2020;30:703–715. 32385659 10.1007/s10895-020-02537-8

[pro70234-bib-0037] McKinney SA , Murphy CS , Hazelwood KL , Davidson MW , Looger LL . A bright and photostable photoconvertible fluorescent protein. Nat Methods. 2009;6:131–133. 19169260 10.1038/nmeth.1296 PMC2745648

[pro70234-bib-0038] Nagai T , Ibata K , Park ES , Kubota M , Mikoshiba K , Miyawaki A . A variant of yellow fluorescent protein with fast and efficient maturation for cell‐biological applications. Nat Biotechnol. 2002;20:87–90. 11753368 10.1038/nbt0102-87

[pro70234-bib-0039] Nienhaus K , Nienhaus GU . Fluorescent proteins of the EosFP clade: intriguing marker tools with multiple photoactivation modes for advanced microscopy. RSC Chem Biol. 2021;2:796–814. 34458811 10.1039/d1cb00014d PMC8341165

[pro70234-bib-0040] Osuga M , Nishimura T , Suetsugu S . Development of a green reversibly photoswitchable variant of Eos fluorescent protein with fixation resistance. Mol Biol Cell. 2021;32:br7. 34495704 10.1091/mbc.E21-01-0044 PMC8693962

[pro70234-bib-0041] Ovesny M , Krizek P , Borkovec J , Svindrych Z , Hagen GM . ThunderSTORM: a comprehensive ImageJ plug‐in for PALM and STORM data analysis and super‐resolution imaging. Bioinformatics. 2014;30:2389–2390. 24771516 10.1093/bioinformatics/btu202 PMC4207427

[pro70234-bib-0042] Paez‐Segala MG , Sun MG , Shtengel G , Viswanathan S , Baird MA , Macklin JJ , et al. Fixation‐resistant photoactivatable fluorescent proteins for CLEM. Nat Methods. 2015;12:215–218. 25581799 10.1038/nmeth.3225 PMC4344411

[pro70234-bib-0043] Pedelacq JD , Cabantous S , Tran T , Terwilliger TC , Waldo GS . Engineering and characterization of a superfolder green fluorescent protein. Nat Biotechnol. 2006;24:79–88. 16369541 10.1038/nbt1172

[pro70234-bib-0044] Pletneva NV , Pletnev VZ , Lukyanov KA , Gurskaya NG , Goryacheva EA , Martynov VI , et al. Structural evidence for a dehydrated intermediate in green fluorescent protein chromophore biosynthesis. J Biol Chem. 2010;285:15978–15984. 20220148 10.1074/jbc.M109.092320 PMC2871466

[pro70234-bib-0045] Pletneva NV , Maksimov EG , Protasova EA , Mamontova AV , Simonyan TR , Ziganshin RH , et al. Amino acid residue at the 165th position tunes EYFP chromophore maturation. A structure‐based design. Comput Struct Biotechnol J. 2021;19:2950–2959. 34136094 10.1016/j.csbj.2021.05.017 PMC8163865

[pro70234-bib-0046] Pouwels LJ , Zhang L , Chan NH , Dorrestein PC , Wachter RM . Kinetic isotope effect studies on the de novo rate of chromophore formation in fast‐ and slow‐maturing GFP variants. Biochemistry. 2008;47:10111–10122. 18759496 10.1021/bi8007164 PMC2643082

[pro70234-bib-0047] Reid BG , Flynn GC . Chromophore formation in green fluorescent protein. Biochemistry. 1997;36:6786–6791. 9184161 10.1021/bi970281w

[pro70234-bib-0048] Rosenow MA , Huffman HA , Phail ME , Wachter RM . The crystal structure of the Y66L variant of green fluorescent protein supports a cyclization‐oxidation‐dehydration mechanism for chromophore maturation. Biochemistry. 2004;43:4464–4472. 15078092 10.1021/bi0361315

[pro70234-bib-0049] Schrodinger, LLC . The PyMOL Molecular Graphics System, Version 1.8. 2015.

[pro70234-bib-0050] Shaner NC , Campbell RE , Steinbach PA , Giepmans BN , Palmer AE , Tsien RY . Improved monomeric red, orange and yellow fluorescent proteins derived from *Discosoma* sp. red fluorescent protein. Nat Biotechnol. 2004;22:1567–1572. 15558047 10.1038/nbt1037

[pro70234-bib-0051] Shaner NC , Lambert GG , Chammas A , Ni Y , Cranfill PJ , Baird MA , et al. A bright monomeric green fluorescent protein derived from *Branchiostoma lanceolatum* . Nat Methods. 2013;10:407–409. 23524392 10.1038/nmeth.2413 PMC3811051

[pro70234-bib-0052] Shinoda H , Ma Y , Nakashima R , Sakurai K , Matsuda T , Nagai T . Acid‐tolerant monomeric GFP from *Olindias formosa* . Cell Chem Biol. 2018;25:330–338. 29290624 10.1016/j.chembiol.2017.12.005

[pro70234-bib-0053] Sniegowski JA , Lappe JW , Patel HN , Huffman HA , Wachter RM . Base catalysis of chromophore formation in Arg96 and Glu222 variants of green fluorescent protein. J Biol Chem. 2005;280:26248–26255. 15888441 10.1074/jbc.M412327200

[pro70234-bib-0054] Sniegowski JA , Phail ME , Wachter RM . Maturation efficiency, trypsin sensitivity, and optical properties of Arg96, Glu222, and Gly67 variants of green fluorescent protein. Biochem Biophys Res Commun. 2005;332:657–663. 15894286 10.1016/j.bbrc.2005.04.166

[pro70234-bib-0055] Sreenivasan VKA , Graus MS , Pillai RR , Yang Z , Goyette J , Gaus K . Influence of FRET and fluorescent protein maturation on the quantification of binding affinity with dual‐channel fluorescence cross‐correlation spectroscopy. Biomed Opt Express. 2020;11:6137–6153. 33282480 10.1364/BOE.401056 PMC7687962

[pro70234-bib-0056] Strack RL , Strongin DE , Bhattacharyya D , Tao W , Berman A , Broxmeyer HE , et al. A noncytotoxic DsRed variant for whole‐cell labeling. Nat Methods. 2008;5:955–957. 18953349 10.1038/nmeth.1264 PMC4107390

[pro70234-bib-0057] Subach OM , Patterson GH , Ting LM , Wang Y , Condeelis JS , Verkhusha VV . A photoswitchable orange‐to‐far‐red fluorescent protein, PSmOrange. Nat Methods. 2011;8:771–777. 21804536 10.1038/nmeth.1664 PMC3164916

[pro70234-bib-0058] Tsien RY . The green fluorescent protein. Annu Rev Biochem. 1998;67:509–544. 9759496 10.1146/annurev.biochem.67.1.509

[pro70234-bib-0059] von Stetten D , Carpentier P , Flot D , Beteva A , Caserotto H , Dobias F , et al. ID30A‐3 (MASSIF‐3)—a beamline for macromolecular crystallography at the ESRF with a small intense beam. J Synchrotron Radiat. 2020;27:844–851. 32381789 10.1107/S1600577520004002 PMC7206554

[pro70234-bib-0060] Vonrhein C , Flensburg C , Keller P , Sharff A , Smart O , Paciorek W , et al. Data processing and analysis with the autoPROC toolbox. Acta Crystallogr D Biol Crystallogr. 2011;67:293–302. 21460447 10.1107/S0907444911007773 PMC3069744

[pro70234-bib-0061] Wachter RM . Chromogenic cross‐link formation in green fluorescent protein. Acc Chem Res. 2007;40:120–127. 17309193 10.1021/ar040086r

[pro70234-bib-0062] Ward WW . Properties of the coelenterate green‐fluorescent proteins. In: DeLuca MA , McElroy WD , editors. Bioluminescence and chemiluminescence: basic chemistry and analytical applications. New York: Academic Press; 1981. p. 235–242.

[pro70234-bib-0063] Wazawa T , Noma R , Uto S , Sugiura K , Washio T , Nagai T . A photoswitchable fluorescent protein for hours‐time‐lapse and sub‐second‐resolved super‐resolution imaging. Microscopy (Oxf). 2021;70:340–352. 33481018 10.1093/jmicro/dfab001 PMC8350982

[pro70234-bib-0064] Wood TI , Barondeau DP , Hitomi C , Kassmann CJ , Tainer JA , Getzoff ED . Defining the role of arginine 96 in green fluorescent protein fluorophore biosynthesis. Biochemistry. 2005;44:16211–16220. 16331981 10.1021/bi051388j

[pro70234-bib-0065] Wulffele J , Thedie D , Glushonkov O , Bourgeois D . mEos4b photoconversion efficiency depends on laser illumination conditions used in PALM. J Phys Chem Lett. 2022;13:5075–5080. 35653150 10.1021/acs.jpclett.2c00933

[pro70234-bib-0066] Zhang H , Lesnov GD , Subach OM , Zhang W , Kuzmicheva TP , Vlaskina AV , et al. Bright and stable monomeric green fluorescent protein derived from StayGold. Nat Methods. 2024;21:657–665. 38409224 10.1038/s41592-024-02203-y PMC11852770

[pro70234-bib-0067] Zhang L , Patel HN , Lappe JW , Wachter RM . Reaction progress of chromophore biogenesis in green fluorescent protein. J Am Chem Soc. 2006;128:4766–4772. 16594713 10.1021/ja0580439

[pro70234-bib-0068] Zhang M , Chang H , Zhang Y , Yu J , Wu L , Ji W , et al. Rational design of true monomeric and bright photoactivatable fluorescent proteins. Nat Methods. 2012;9:727–729. 22581370 10.1038/nmeth.2021

[pro70234-bib-0069] Zhang M , Fu Z , Li C , Liu A , Peng D , Xue F , et al. Fast super‐resolution imaging technique and immediate early nanostructure capturing by a photoconvertible fluorescent protein. Nano Lett. 2020;20:2197–2208. 31576756 10.1021/acs.nanolett.9b02855

